# Knowledge, attitudes and behaviors on antimicrobial resistance among general public across 14 member states in the WHO European region: results from a cross-sectional survey

**DOI:** 10.3389/fpubh.2023.1274818

**Published:** 2023-11-23

**Authors:** Sauman Singh-Phulgenda, Pantelis Antoniou, Danilo Lo Fo Wong, Kotoji Iwamoto, Ketevan Kandelaki

**Affiliations:** ^1^Infectious Diseases Data Observatory (IDDO), University of Oxford, Oxford, United Kingdom; ^2^WHO Regional Office for Europe, Copenhagen, Denmark

**Keywords:** antimicrobial resistance (AMR), antibiotics, KAB survey, WHO European region, behavior, antibiotic resistance

## Abstract

**Background:**

Antimicrobial resistance (AMR) is a major global public health threat requiring urgent action. Pan-European data on knowledge, attitudes and behaviors among the general public regarding antibiotic use and AMR is limited.

**Methods:**

A multicentric, cross-sectional survey of the general public was conducted in the capital cities of 14 Member States of the WHO European Region. A validated questionnaire from the AMR Eurobarometer survey was used to collect data on antibiotic use and knowledge, access to antibiotics, and understanding of policy responses through face-to-face exit interviews.

**Results:**

Out of 8,221 respondents from 14 Member States, 50% took antibiotics in the past 12 months and the majority (53%) obtained their most recent course from a medical practitioner. The most reported reasons for taking antibiotics orally in the past 12 months were cold (24%), sore throat (21%), cough (18%), and flu (16%). Overall, 84% of participants showed a lack of knowledge about appropriate antibiotic use. However, only 37% of respondents reported receiving any information in the past year about the importance of avoiding unnecessary antibiotic use. Doctors were the most cited (50%) and most trusted (80%) source of information. Among respondents who experienced COVID-19, 28% took antibiotics with a prescription, while 8% took antibiotics without a prescription.

**Conclusion:**

This study highlights the urgent need for targeted awareness campaigns and educational initiatives to address knowledge gaps and promote responsible antibiotic use. The findings emphasize the role of the general population in combating AMR. The data serve as baseline information for future evaluations and interventions in the Region.

## Introduction

Antimicrobial resistance (AMR) is recognized by the WHO as one of the ten major global public health threats (1). AMR occurs when microorganisms develop mechanisms to resist the effects of antimicrobial drugs that are typically used to treat infections (1, 2). There are different types of antimicrobials, such as antibiotics for bacteria, antivirals for viruses, and antifungals for fungi, each targeting specific types of microorganisms. While AMR is a natural phenomenon, its development and spread is accelerated by antibiotic use, rendering infections more challenging to treat effectively (2, 3).

Several factors contribute to the development and spread of AMR including: the misuse and overuse of antimicrobials in the human health, veterinary and agricultural sectors; inadequate access to clean water, sanitation and hygiene for both humans and animals; suboptimal infection prevention and control practices in health-care facilities and farms; limited availability of quality, affordable medicines, vaccines and diagnostics; inadequate awareness and knowledge among health-care providers and the public; and inadequate enforcement of legislation to regulate antimicrobial use (4–6). A 2022 study in the Lancet estimated 4.95 million deaths associated with bacterial AMR in 2019 worldwide (7). Likewise, more than 35 000 people reportedly die from antimicrobial-resistant infections in the European Union and European Economic Area (EU/EEA) annually while another publication estimated 541 000 deaths associated with bacterial AMR and 133 000 deaths attributable to bacterial AMR in the WHO European Region in 2019 (8, 9). Multidrug-resistant strains of pathogens are increasing in hospital settings, and the spread of antimicrobial resistant infections in community settings can be accelerated by various geopolitical, financial, and sociocultural factors (1, 10). Addressing the inappropriate use of antimicrobials in community settings is one of the key components in the fight against AMR.

Indeed, two of the five objectives in the Global Action Plan on Antimicrobial Resistance are “to improve awareness and understanding of antimicrobial resistance through effective communication, education and training” and “to optimize the use of antimicrobial agents” (11). AMR is affected by a complex set of behavioral factors including overprescribing, self-medication, over-the-counter (OTC) sales, as well as overuse in animal husbandry, making it difficult to ascertain a primary cause or actor. In human medicine, outpatient settings account for the majority of antibiotic use, either prescribed by clinicians or obtained without valid prescription or a doctor's consultation (12). OTC sale of antibiotics, without prescription, is a major challenge contributing to inappropriate antibiotic use in the community in many countries globally, including in the WHO European Region (the Region) and the situation is further exacerbated by use of antibiotics in agriculture (13–15). A recent systematic review and meta-analysis reported that, across 38 studies from 24 countries, the pooled proportion of non-prescription supply of antibiotics was 62% (12). In such settings where the health systems are weak and legislation is not well enforced, the general public is a key player to bring a change to improve antibiotic use.

There are several quantitative and qualitative studies conducted to investigate public knowledge, attitudes, and behaviors (KAB) on antibiotic use and AMR, mainly among EU/EEA Member States in the Region and sporadically among some other Member States in the Region (16–23). Since 2009, the European Commission (EC) has administered a periodic survey among the general public in EU/EEA Member States to monitor the levels of usage of, and knowledge about, antibiotics and most recently conducted the survey in 2022 (24).

Since 2015, the WHO Regional Office for Europe has been supporting its Member States to mark World Antimicrobial Awareness Week, rebranded as World AMR Awareness Week (WAAW, 18–24 November annually), campaigns that build on European Antibiotic Awareness Day (EAAD, 18 November annually, also supported by WHO European Region since 2012). However, an in-depth understanding about the knowledge among general public concerning antibiotic use and AMR as well as impact of information on behavioral change outside of EU/EEA Member States in the Region is limited. There are several quantitative and qualitative studies conducted to investigate public knowledge, attitudes and behaviors (KAB) on antibiotic use and AMR (16–23). However, the primary focus of these studies is medical practitioner. Since 2009, the European Commission has undertaken a survey among the general public in European Union and European Economic Area (EU/EEA) Member States, as part of AMR Eurobarometer surveys, to monitor the levels of usage of, and knowledge about, antibiotics. The last AMR Eurobarometer survey took place in 2022. This periodic survey allows for a comparison of trends and monitoring over time. Despite this, comprehensive evidence across the entire WHO European Region is limited and information, where available, is not collected in a harmonized manner, limiting the cross country/region and even national analysis. Furthermore, a recent report highlighted a concern in antibiotic use for prevention and treatment of COVID-19 across nine Member States of the Region (13, 25).

To address this lack of data, the WHO Regional Office for Europe conducted a survey using the same questionnaire used by the EC for the 2022 Eurobarometer survey with the aims of establishing a harmonized baseline data on KAB on antibiotic use and AMR in 14 WHO European Region Member States. The data are expected to support participating Member States in the development of targeted awareness raising and education interventions, and subsequently the evaluation of their impact.

## Methods

### Study design and setting

This study was designed as a multicentric, cross-sectional survey. Data was collected using a validated questionnaire by trained data collectors through face-to-face exit interviews over a six-week period between 12 October and 17 November 2022 in the capital cities of 14 Member States in the Region, namely: Albania (ALB), Armenia (ARM), Azerbaijan (AZB), Bosnia and Herzegovina (BIH), Belarus (BLR), Georgia (GEO), Kazakhstan (KAZ), Kyrgyzstan (KGZ), Montenegro (MNE), North Macedonia (MKD), Republic of Moldova (MDA), Tajikistan (TJK), Türkiye (TUR) and Uzbekistan (UZB).

### Participants

The target sample in this study were adults aged 18 years or older living in the cities where the survey was conducted who were able to give informed consent. The geographic area was limited primarily to capital cities due to limited resources and time (Supplementary Table 1 for a list of participating countries and respective cities).

### Questionnaire development and implementation

We adapted the same questionnaire that has been developed and used for the Eurobarometer survey by the EC (24). The questionnaire was divided into two sections. The first section consisted of five items on participants' sociodemographic characteristics: gender, age, education, profession, and financial status. The second section consisted of a series of 16 items focusing on respondents' use and knowledge of, and access to antibiotics, including the impact of COVID-19, as well the role of sources of information used for awareness raising, and their understanding of the policy response to tackle AMR.

The English version of the questionnaire acted as the source document and was translated into 14 national languages spoken in the participating countries (Supplementary File 1). Translation from English to specific languages was carried out by translators and expert team members from the participating countries.

KoBoToolbox, a free open-source suite of tools for mobile data collection, was used for implementing the questionnaire using android-based tablets in the field by trained data collectors in each country. All data collectors received intensive training of the study protocol, including the questionnaire, informed consent, and interview methodology. Initially, the translated versions of the survey was validated by the leads and data collectors in each country through a pilot run. Any issues identified with the data collection tool were resolved prior to initiating the actual survey.

This survey was designed to collect the baseline information in the participating countries and not to detect any specific effect sizes. A minimum sample size of 385 respondents with complete interviews was required to achieve a 95% confidence level with a 5% of margin of errors for a population of 5 million (the largest population of any participating capital city in this survey). For convenience and uniformity across the participating countries it was decided to set the sample-size to 500 to ensure completeness. From each participating city, the country leads created a sampling frame that listed of 28 most visited potential survey sites for each city for exit interviews. The sampling frame consisted of the following locations: (i) metro, bus or train stations; (ii) shopping malls; (iii) hospitals; (iv) universities; and (v) pharmacies as applicable in each of the selected cities. An independent member used a random number generator in Microsoft Excel to randomly select 10 sites for each city from the sampling frame. In each country, data was collected simultaneously at all sites during the survey period with a target of a minimum of 50 interviews per site.

Furthermore, a systematic random sampling approach was applied to select study participants as follows: every alternate person exiting the survey site (for each site, an exit point was fixed for reference) was approached, and if the person interacted, the data collectors assessed eligibility and explained the purpose and objectives of the survey to the potential participant. They were also informed about their anonymity if they participated, and the contact information of the country study coordinator was shared if they requested further information.

### Ethical considerations

Oral informed consent was sought from each participant before starting each interview (Supplementary File 1). The questionnaire was not administered if a participant did not meet eligibility criteria or refused to grant informed consent. The study was confirmed as exempted from review by the WHO Ethics Review Committee (Protocol Number ERC.0003790).

### Data collection and analysis

After the data was downloaded from KoBoToolbox, it was cleaned, re-coded, and prepared for analysis using Microsoft Excel (Microsoft, Redmond, Washington). A descriptive analysis was performed for all variables, as frequencies, percentages and items concerning respondents' KAB were tabulated and graphed. Sub-group analysis was performed at the country level to get better insight from the available data.

## Results

### Demography

A total of 9,602 participants were approached for interview and 8,221 (86%) respondents from 14 Member States provided informed consent and participated in the survey (Supplementary Table 2). Each participating Member State reached the minimum sample size of 500 interviews. Overall, 57% of the 8,221 respondents across all participants identified as females and 42% identified as males. Over 60% of the participants were <40 years-old and most of them completed their studies in their 20s (47%). Nearly a quarter of the participants were either office workers (25%) or students (23%). Close to one out of ten participants were either engaged in manual work (10%) or were self-employed (14%) and another 8% were retired. Forty percent of the participants faced difficulty in paying their bills from time to time, followed by another 20% who faced difficulty in paying their bills most of the time (Table 1).

**Table 1:** Sociodemographic characteristics of survey participants.

	***n***	**%**
**Gender (*****N*** = **8,221)**		
Female	4,709	57.28%
Male	3,469	42.20%
Other	26	0.32%
Do not wish to answer	17	0.21%
**Age (*****N*** = **8,221)**		
18–24	2,297	27.94%
25–39	2,786	33.89%
40–54	1,817	22.10%
55+	1,321	16.07%
**Age at the end of education (*****N*** = **8,221)**		
<=15	335	4.07%
16–19	2,063	25.09%
20+	3,904	47.49%
Still studying	1,919	23.34%
**Socio-professional category (*****N*** = **8,221)**		
Other white collar office worker	2,065	25.12%
Students	1,885	22.93%
Self-employed	1,131	13.76%
Manual workers	840	10.22%
Retired	709	8.62%
Managers	537	6.53%
House persons	531	6.46%
Unemployed	523	6.36%
**Difficulty in paying bills (*****N*** = **8,221)**		
Most of the time	1,606	19.54%
From time to time	3,309	40.25%
Almost never	1,872	22.77%
Never	1,434	17.44%

### Use of antibiotics

**Figure 1:**
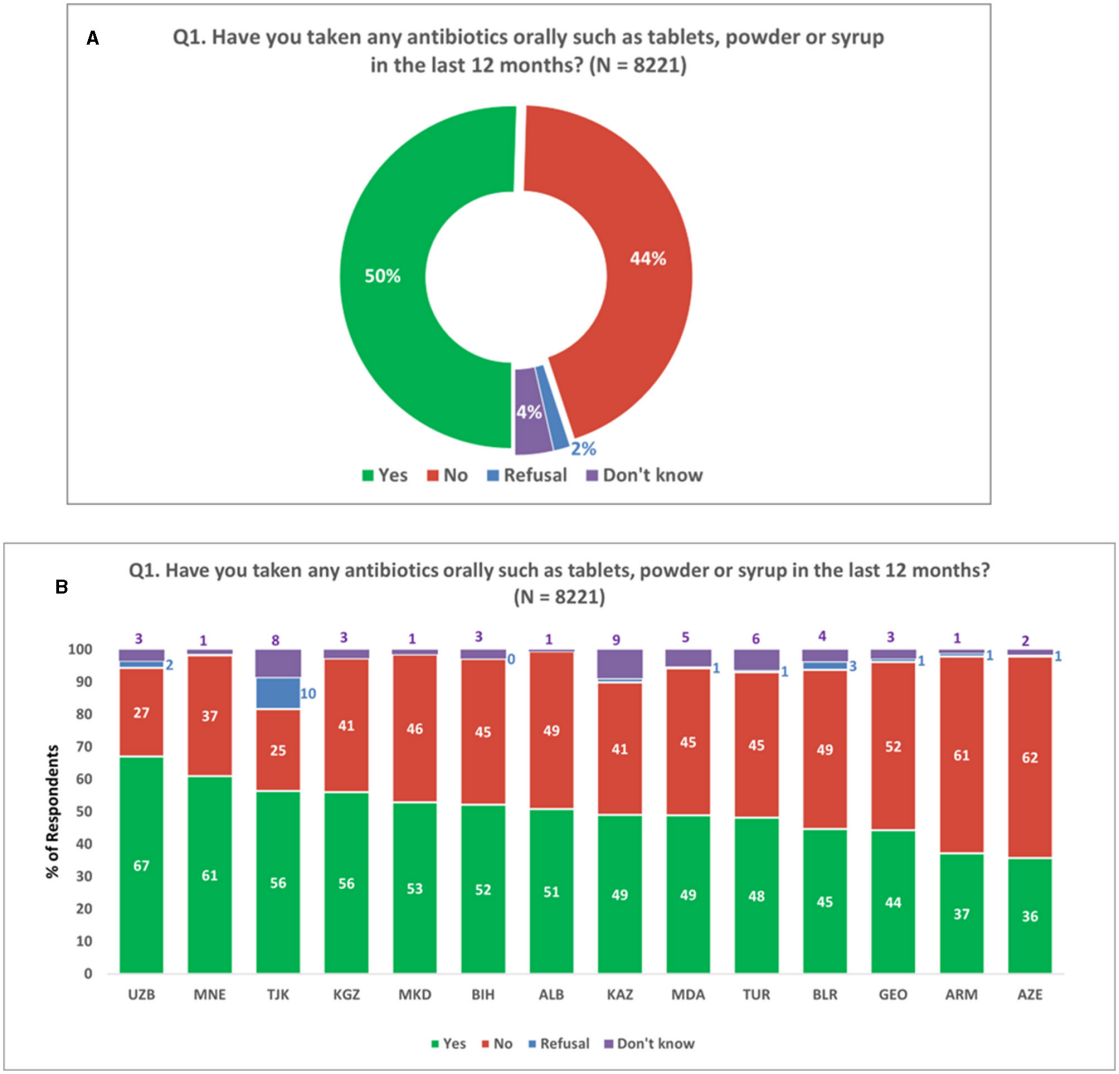
Consumption of oral antibiotics. **(A)** Consumption of oral antibiotics in the last 12 months. **(B)** Consumption of oral antibiotics in last 12 months across survey countries. Respondents were allowed to provide only one response for Q1.

A subset of four questions (Q1 to Q4 in Supplementary File 1) in the survey was designed to help understand the use of antibiotics among the survey population by asking whether they have used antibiotics in the last year, how they obtained them, the reason for taking them, and if they used antibiotics following a diagnosis. Fifty percent of all respondents (*N* = 8,221) had taken antibiotics orally in the last 12 months (Figure 1A). In participating Member States, this ranged from 36 to 67% (Figure 1B).

**Figure 2:**
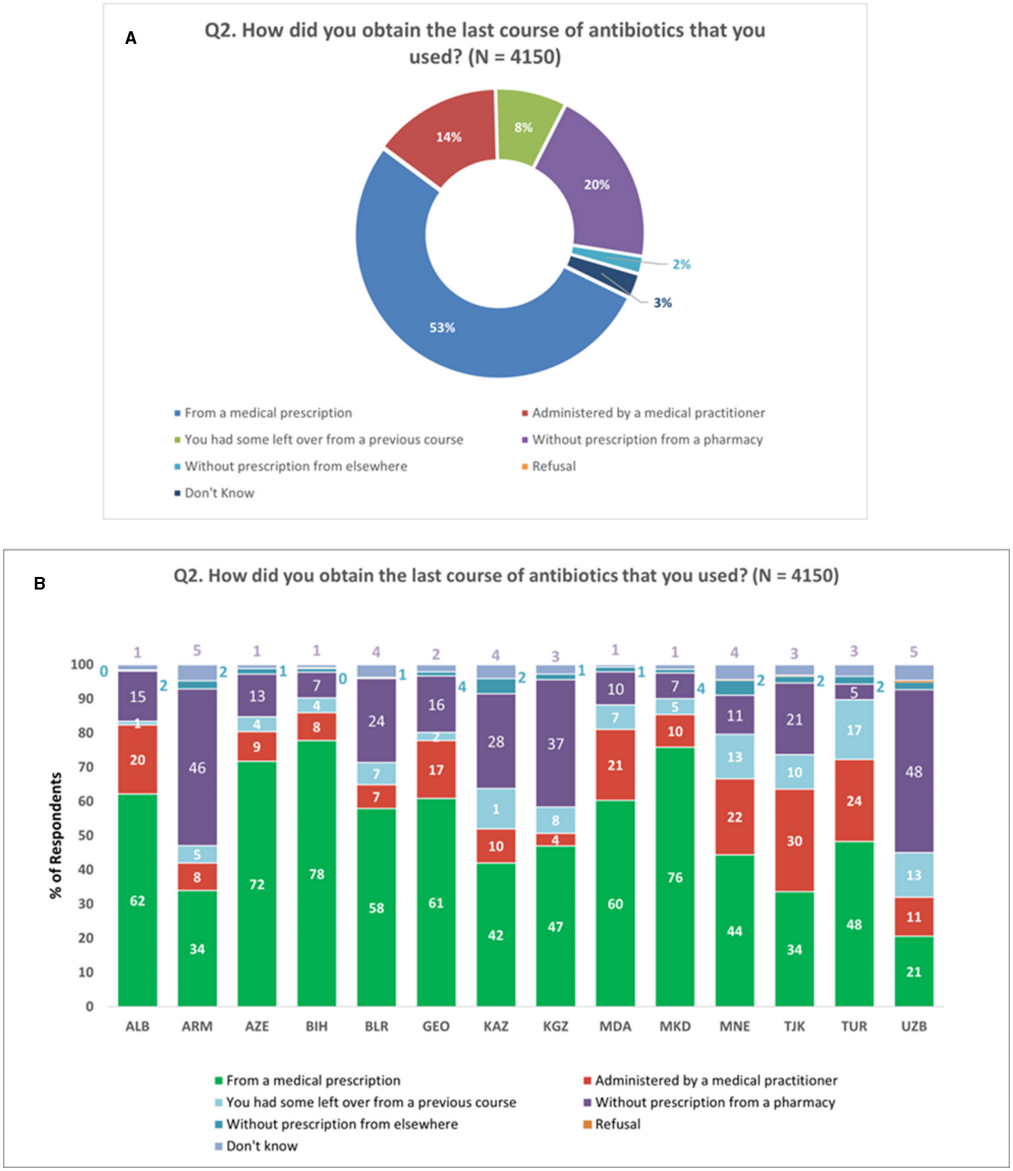
Antibiotic source. **(A)** Source of the last course of antibiotics. **(B)** Source of the last course of antibiotics at national level. Respondents were allowed to provide only one response for Q2.

Among the respondents who had taken antibiotics orally within the past 12 months (*N* = 4,150), over a fifth (22%) obtained oral antibiotic formulations without a prescription. Additionally, 8% of the respondents reported using leftover antibiotics from a previous course. The majority of respondents (53%) obtained their most recent course of antibiotics from a medical practitioner, while 14% had antibiotics administered by a medical practitioner (Figure 2A). Notably, there was considerable variation in respondents' answers at the national level, indicating heterogeneity in antibiotic sourcing practices (Figure 2B).

**Figure 3:**
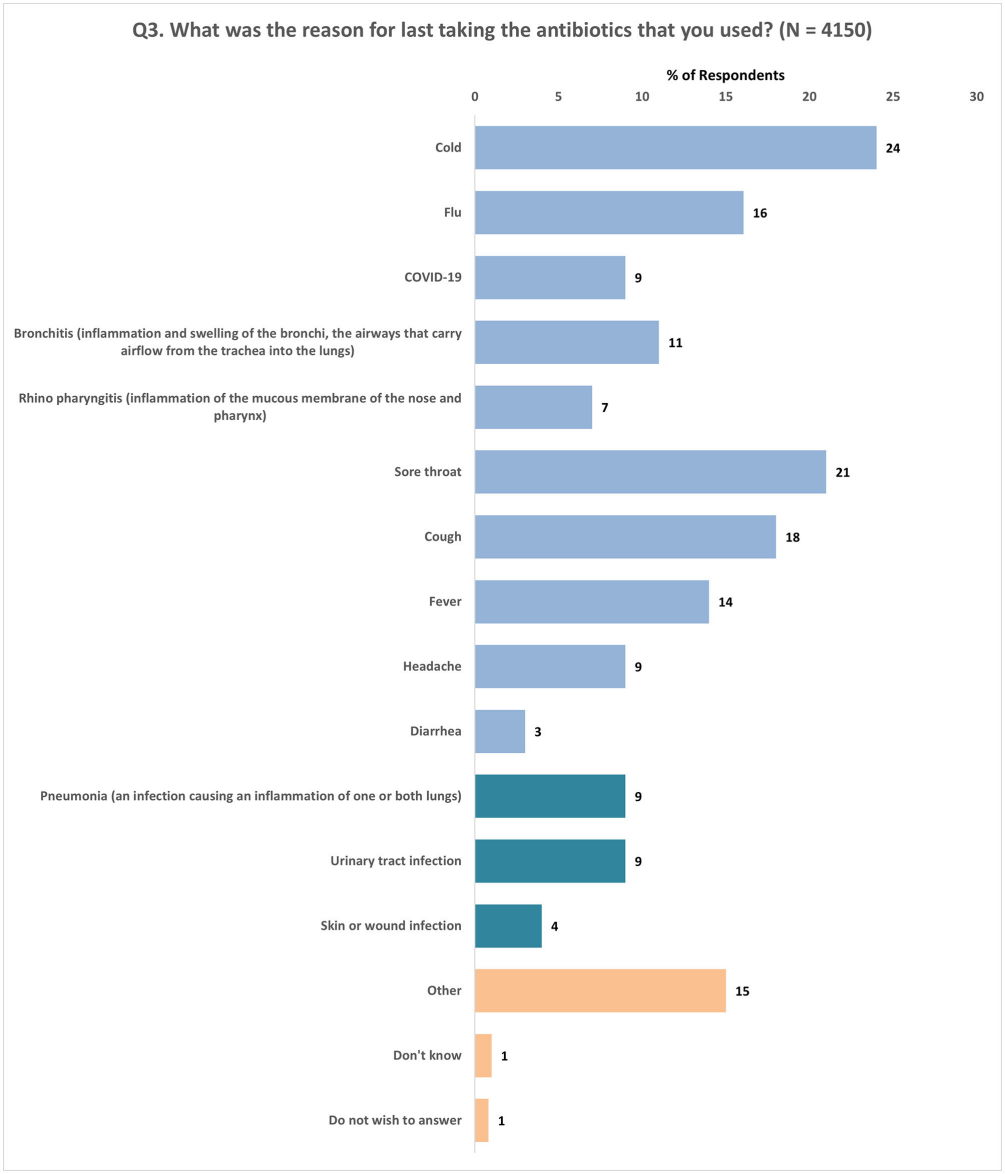
Reasons cited for taking antibiotics. Respondents were allowed to provide multiple responses for Q3.

Regarding the reasons for taking antibiotics among those who had used them orally in the past 12 months (*N* = 4,150), the most commonly reported reasons were cold symptoms (24%), sore throat (21%), cough (18%), and flu-like symptoms (16%). Urinary tract infections and pneumonia were each cited by 9% of respondents (Figure 3).

Next, respondents who reported to have taken antibiotics orally in the last 12 months were asked if they had taken a test to find out the cause of illness, before or at the time of starting antibiotics. The findings revealed that less than half (47%) of the respondents had undergone diagnostic testing before or at the same time as starting antibiotics. In contrast, 45% stated that they had not undergone any testing (Supplementary Figure 1A). Notably, the analysis at the national level showcased considerable variation in diagnostic testing prior to antibiotic use, ranging from 31 to 67% (Supplementary Figure 1B).

### Knowledge of antibiotics

**Figure 4:**
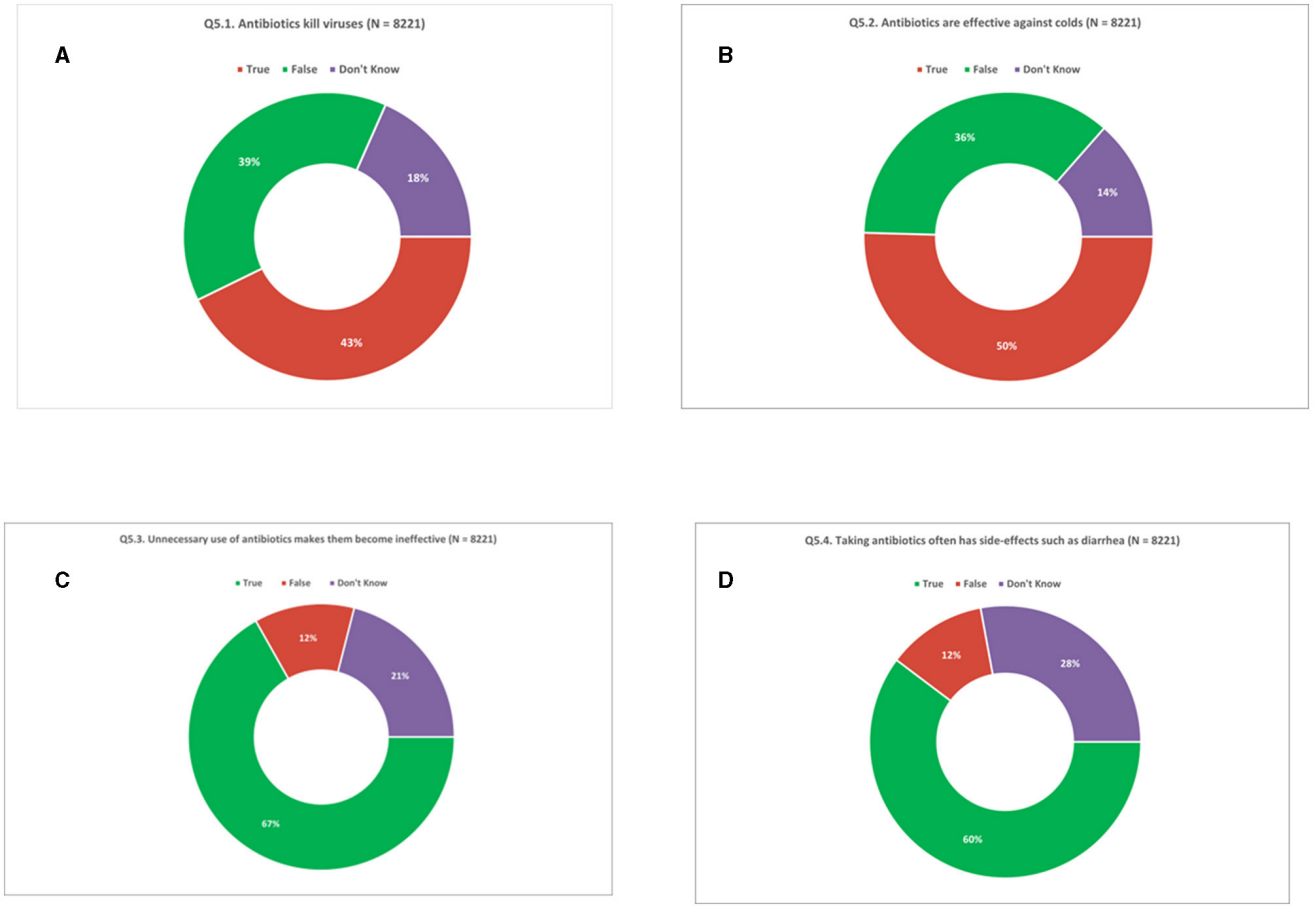
Knowledge of antibiotics. **(A)** Antibiotics kill viruses. **(B)** Antibiotics are effective against colds. **(C)** Unnecessary use of antibiotics makes them become ineffective. **(D)** Taking antibiotics often has side-effects such as diarrhea. Respondents were allowed to provide only one response for Q5.1 to Q5.4.

In question 5 (Supplementary File 1), respondents (*N* = 8,221) were presented with a set of four statements to assess their knowledge about the use of antibiotics. They were asked to indicate whether each statement was “True” or “False” or to select “Don't know.” Across all participants (*N* = 8,221), 43% incorrectly thought that it is true that “antibiotics kill viruses,” whereas 39% of the respondents correctly reported that the statement is false. Nearly one-fifth (18%) of the respondents were unable to express an opinion (Figure 4A). Similarly, when asked if “antibiotics are effective against colds,” 50% of the respondents incorrectly said that it is true that antibiotics are effective against colds, while 36% correctly thought that the statement was false. Fourteen percent of the respondents were unable to express an opinion (Figure 4B).

Two-thirds (67%) of the respondents correctly thought it to be true that “unnecessary use of antibiotics makes them become ineffective” whereas 12% incorrectly thought it to be false and one in five respondents (21%) did not have an opinion (Figure 4C). Furthermore, three in five respondents (60%) correctly thought that “antibiotics lead to side effects” whereas 12% incorrectly thought the statement to be false. Over a quarter (28%) of respondents were unable to provide an answer (Figure 4D). In fact, only 16% of respondents were able to correctly validate all four statements. Conversely, 15% of respondents could not correctly validate any of the statements (Supplementary Figures 2E, F).

**Figure 5:**
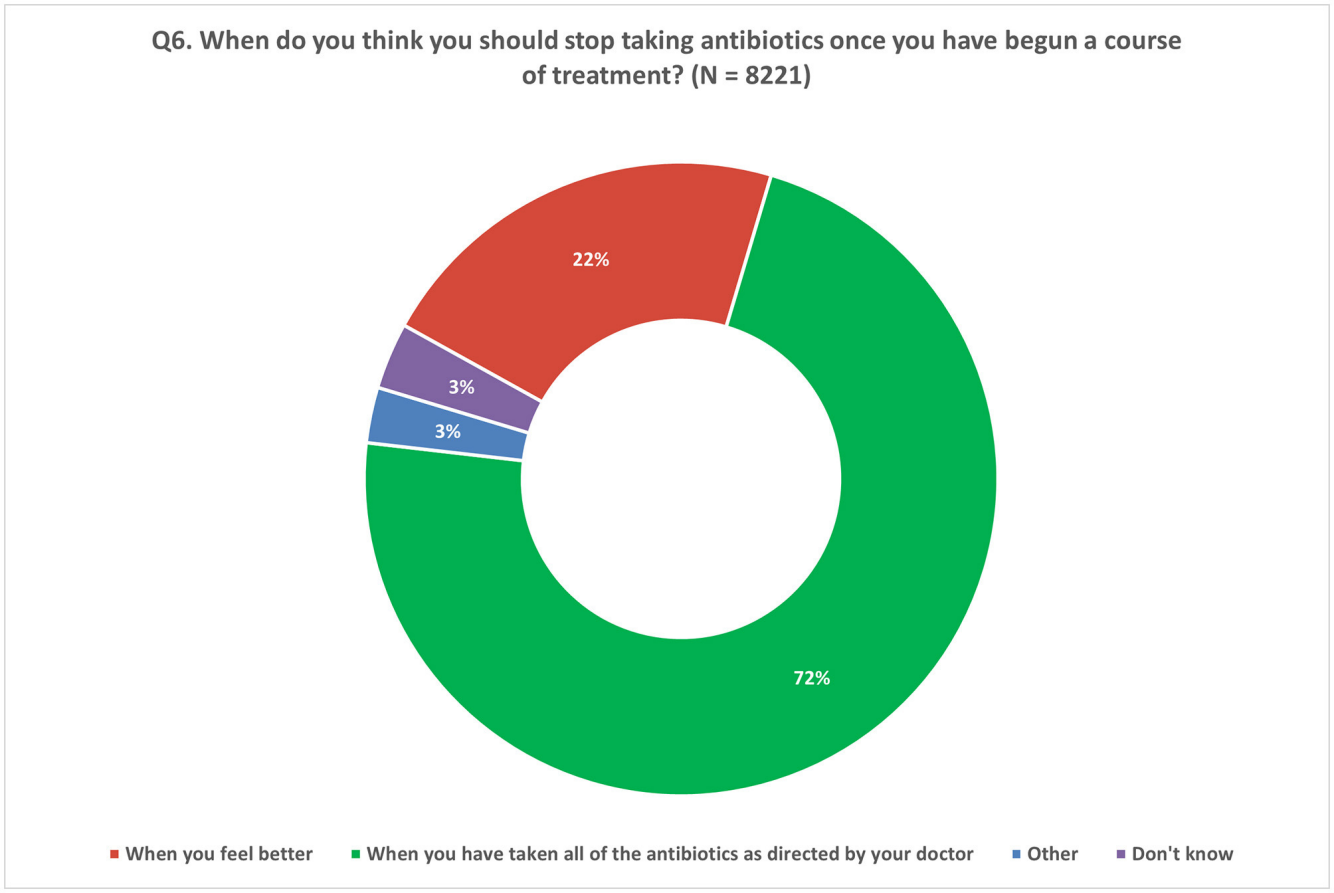
Understanding of compliance to recommended treatment. Respondents were allowed to provide only one response for Q6.

When asked about the appropriate duration of antibiotic treatment, a majority of participants (72%) correctly emphasized the importance of completing the full course as directed by a doctor (Figure 5). However, a notable proportion (22%) held the misconception that antibiotics can be stopped once they start feeling better (Figure 5). The trend of acknowledging the significance of completing the prescribed antibiotic course ranged from 56 to 89% among participating Member States (Supplementary Figure 3).

### Information about the correct use of antibiotics

To evaluate participants' access to information regarding the unnecessary use of antibiotics, a series of six questions (Q7 to Q12, Supplementary File 1) was administered. These questions aimed to assess participants' recent acquisition of knowledge on this topic, identify the sources from which they obtained information, and examine its influence on their antibiotic consumption behavior. Additionally, Q12 explored participants' perceptions and attitudes on the credibility of the information source.

**Figure 6:**
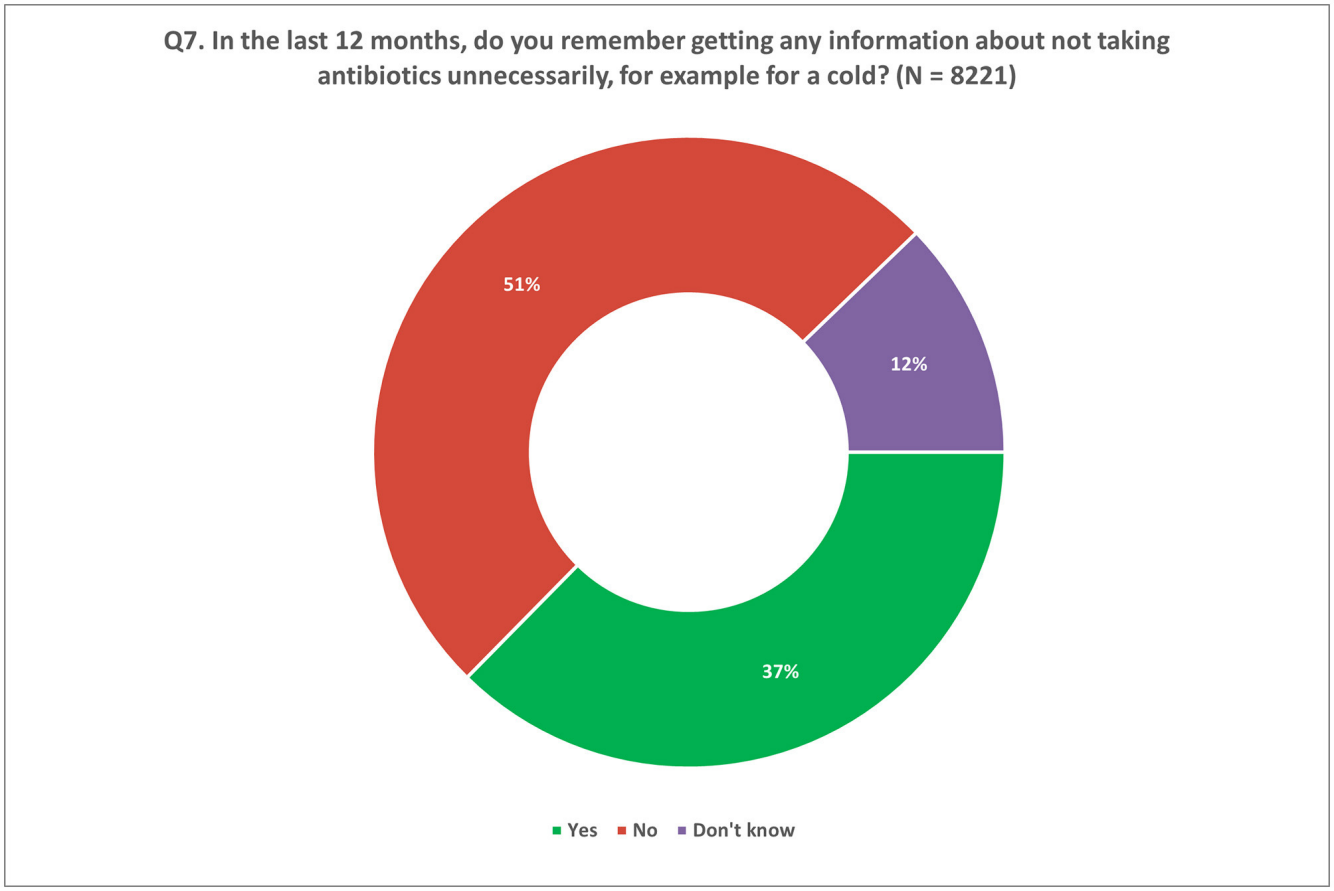
Information received by participants on unnecessary use of antibiotics in the last 12 months. Respondents were allowed to provide only one response for Q7.

Only 37% of respondents reported receiving any information in the past 12 months regarding the importance of avoiding unnecessary antibiotic use. Surprisingly, over half of the respondents (51%) denied receiving any information on the topic, while a smaller portion (12%) indicated they were unsure (Figure 6). These findings were consistent across participating Member States, with response rates ranging from 23 to 48% reporting no exposure to information about unnecessary antibiotic use in the past year (Supplementary Figure 4).

**Figure 7:**
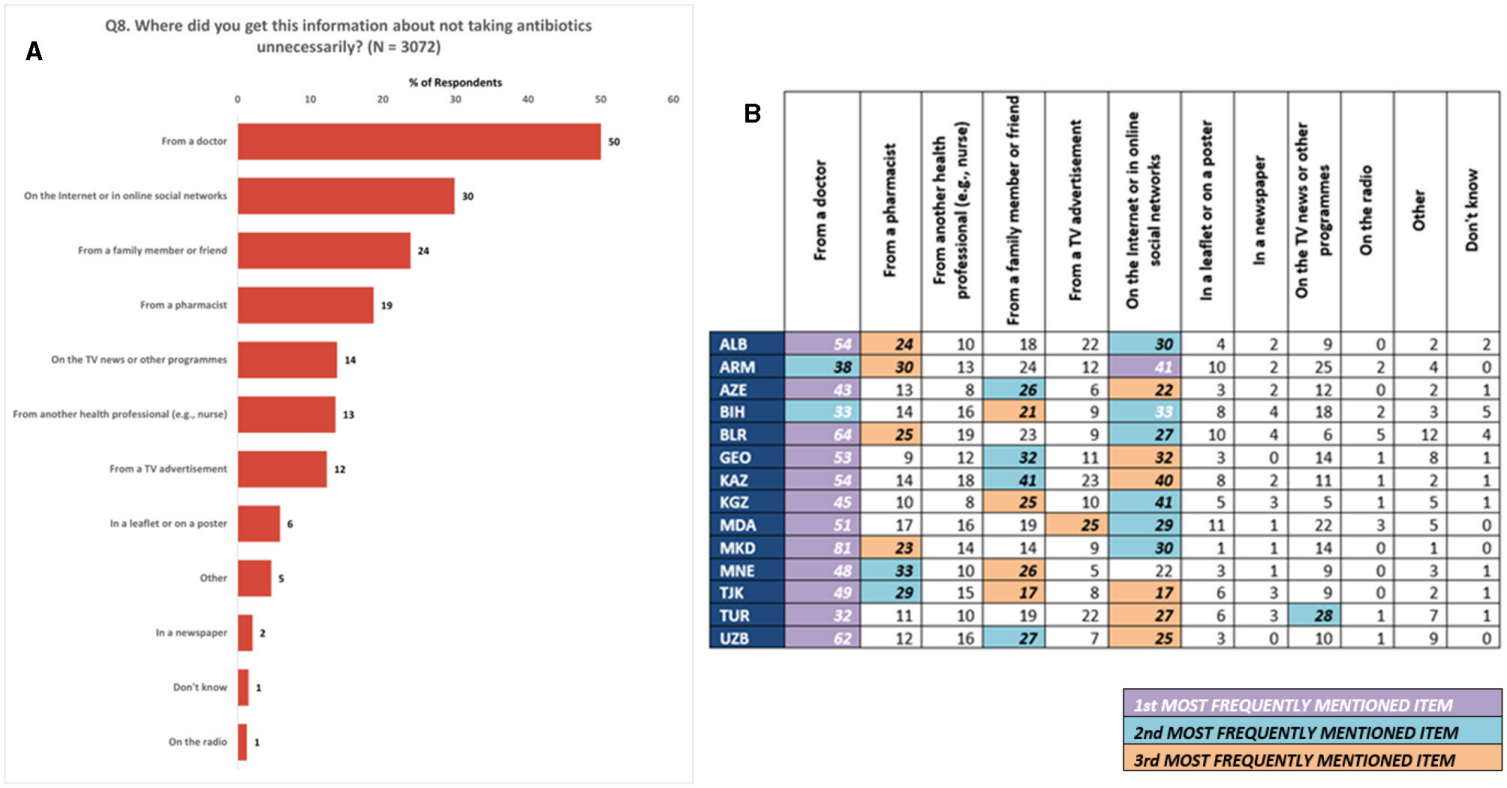
Information sources. **(A)** Most frequent sources of information on unnecessary antibiotics use in the last 12 months. **(B)** Most frequent sources of information on unnecessary antibiotics use in the last 12 months at national level. (1) All numbers in **(B)** are percentages of respondents. (2) Respondents were allowed to provide multiple responses for Q8.

Participants who indicated receiving information about the unnecessary use of antibiotics in the past 12 months (responding “Yes” to question 7; *N* = 3,072) were further queried about the sources of this information. They were presented with a list of potential sources and asked to select multiple responses. The most commonly cited source of information was doctors at 50%. The internet and social networks, as well as family or friends, were also mentioned, accounting for 30 and 24% of responses, respectively (Figure 7A). These patterns were consistent at the national level, as observed in Figure 7B.

Similarly, among those who reported receiving information about the inappropriate use of antibiotics (*N* = 3,072), 65% expressed that this information would alter their perspectives on antibiotic usage (Supplementary Figure 5A). Conversely, 26% stated that their views on antibiotic use remained unchanged, while 9% responded with uncertainty by selecting they “Don't know.” National level analyses revealed a range of responses (42 to 84%) with agreement that information on unnecessary antibiotic use could influence their views on antibiotics (Supplementary Figure 5B).

**Figure 8:**
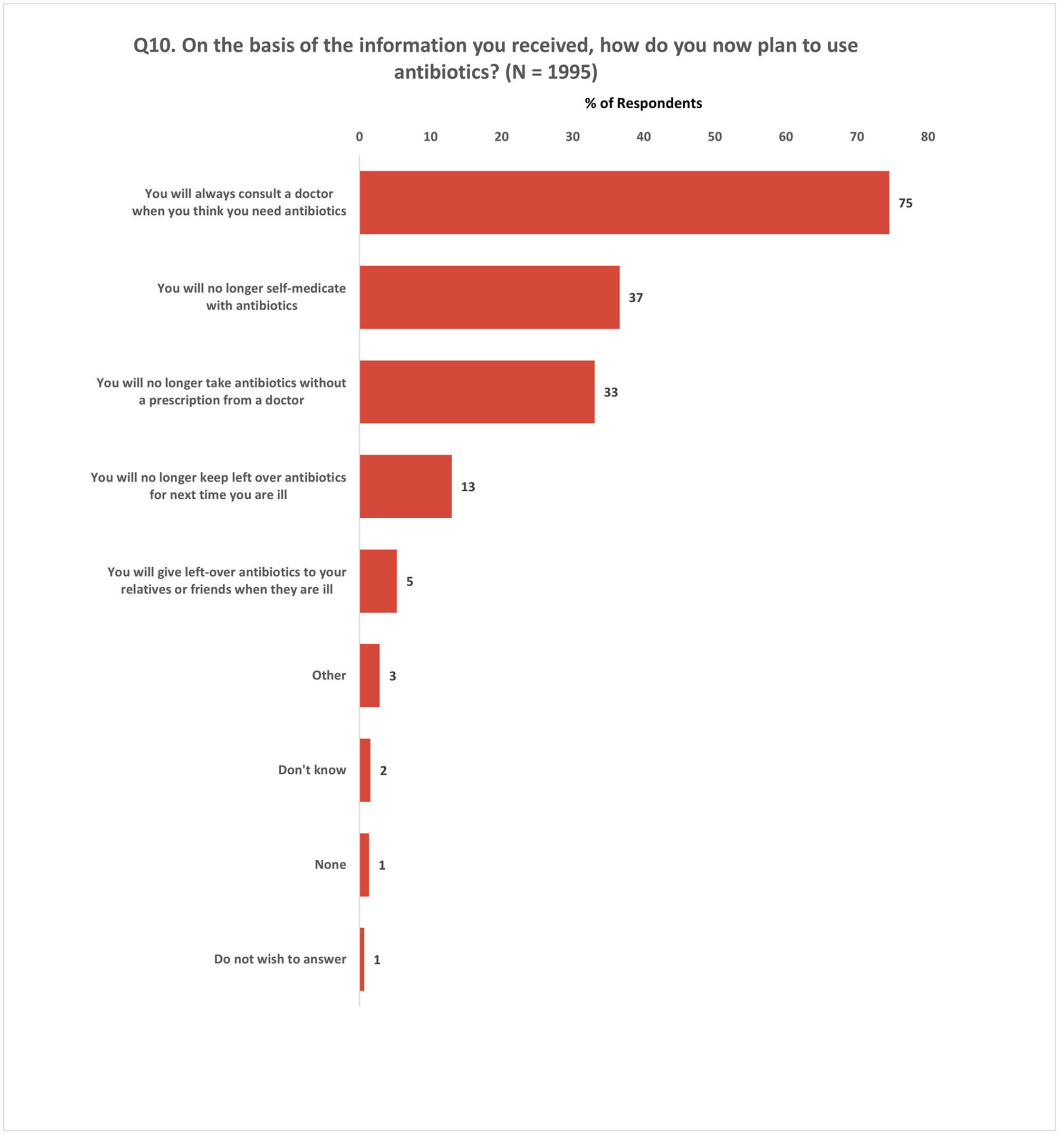
Impact of information on antibiotic use behavior. Respondents were allowed to provide multiple responses for Q10.

Subsequently, participants who indicated that the information they received had influenced their views on antibiotic use (Q9, Supplementary File 1) were asked an additional question (Q10, Supplementary File 1) regarding their intentions for future antibiotic use based on this newfound understanding. Among these respondents (*N* = 1,995), 75% expressed their intention to always consult a doctor when they believe they require antibiotics. Additionally, 37% stated that they would refrain from self-medication, while 33% indicated their decision to avoid obtaining antibiotics without a prescription. Merely 5% of respondents mentioned their inclination to give leftover antibiotics to relatives or friends (Figure 8).

**Figure 9:**
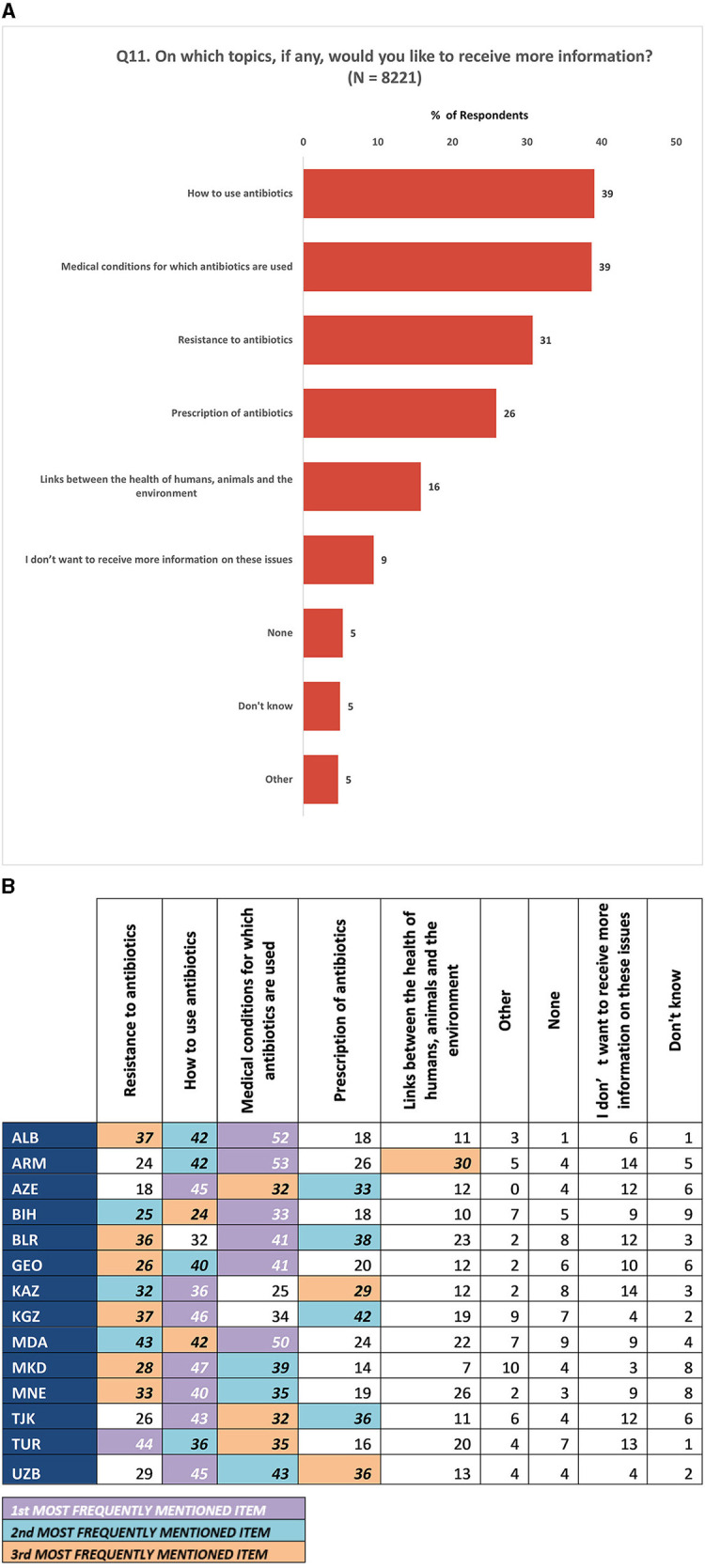
**(A)** Topics for which the participants expressed a desire to receive more information. (**B)** Topics for which the participants expressed a desire to receive more information at national level. (1) All numbers are percentages of respondents. (2) Respondents were allowed to provide multiple responses for Q11.

Inquiring about the specific areas in which respondents (*N* = 8,221) desired more information, it was found that 39% expressed their interest in receiving additional knowledge about the proper use of antibiotics and the medical conditions for which antibiotics are prescribed. Additionally, close to one-third (31%) of the survey participants indicated their curiosity in acquiring further understanding about resistance to antibiotics (Figure 9A). Notably, these topics emerged as the top three most frequently identified areas of interest among participants across all Member States (Figure 9B).

**Figure 10:**
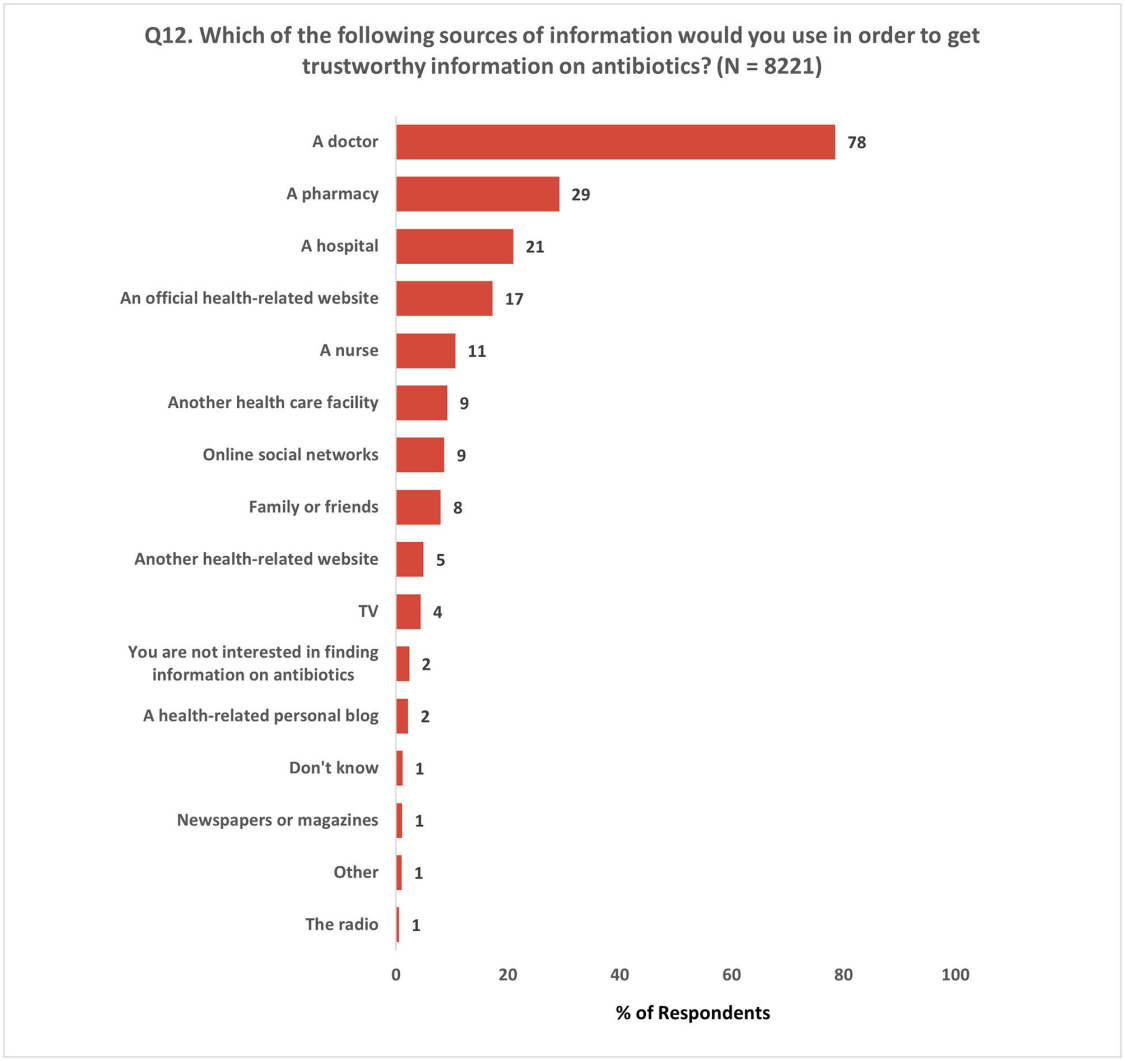
Most relied sources of trustworthy information on antibiotics. Respondents were allowed to provide a maximum of three responses for Q12.

In the final question (Q12) of this section, participants were asked to select three sources they would rely on to obtain trustworthy information about antibiotics. The results showed that ~80% of participants, considered doctors to be the most reliable source for antibiotic-related information. In addition, participants expressed trust in pharmacies (29%), hospitals (21%), and nurses (11%). Furthermore, 17% of the respondents reported placing their confidence in official health-related websites (Figure 10).

### Impact of COVID-19 on antibiotic usage and access

**Figure 11:**
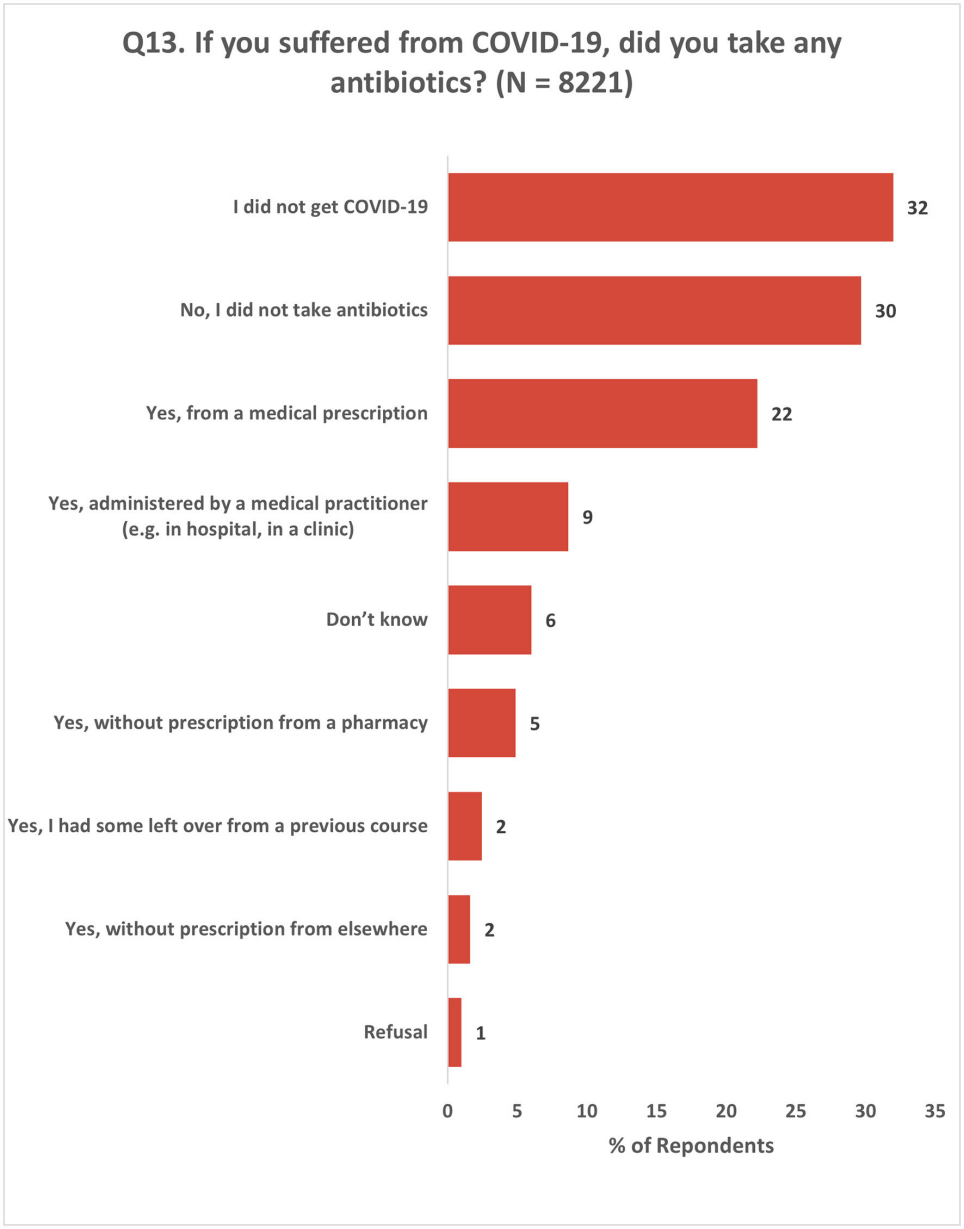
Use of antibiotics by the respondents for COVID-19. Respondents were allowed to provide multiple responses for Q13.

This section included two questions that focused on the impact of COVID-19 on the use of and access to antibiotics among the survey respondents. Among those who reported having COVID-19, 30% did not take antibiotics, while 28% took antibiotics with a prescription and 8% took antibiotics without a prescription (Figure 11; Supplementary Figure 6).

**Figure 12:**
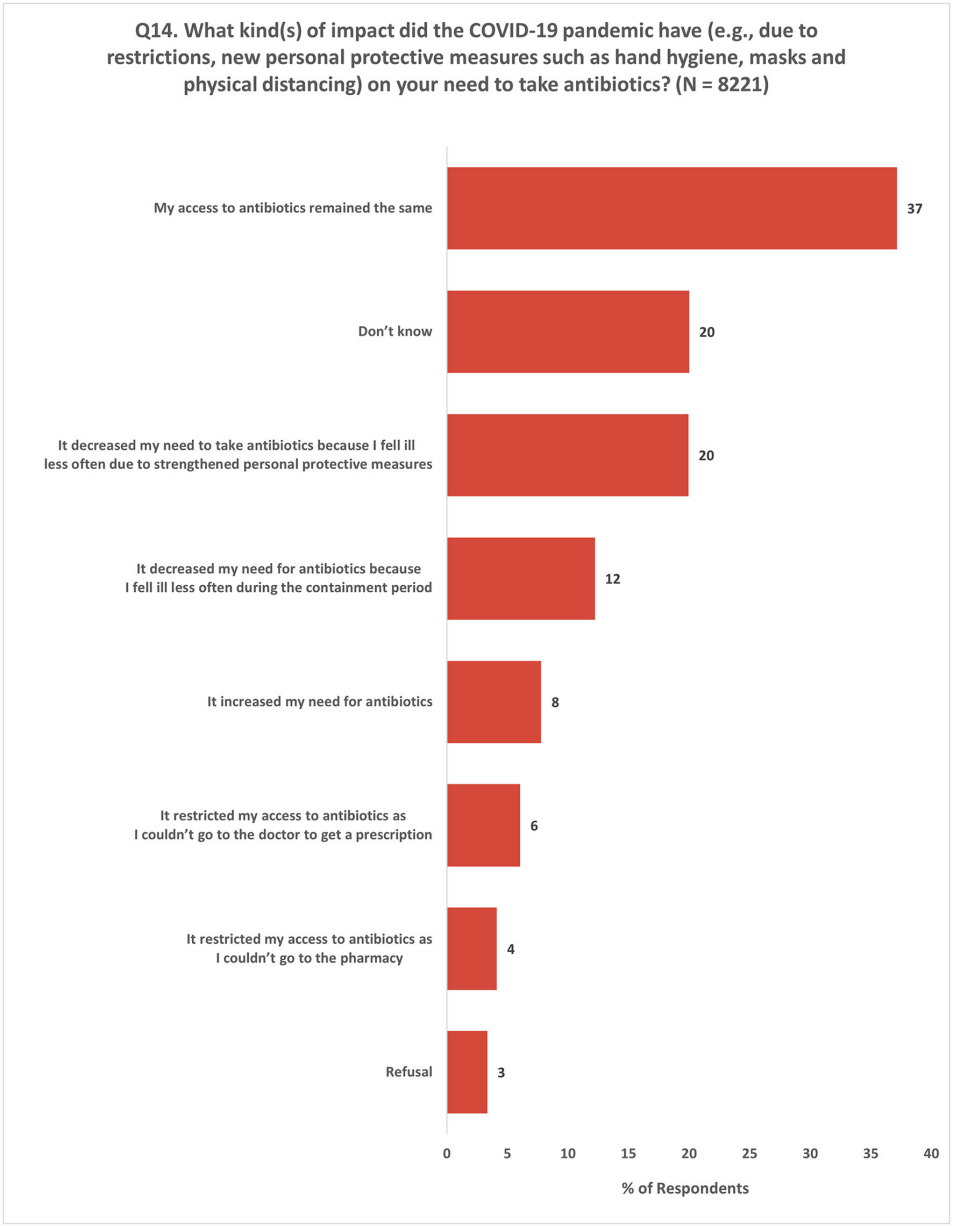
Impact of COVID-19 on the need to take antibiotics. Respondents were allowed to provide multiple responses for Q14.

The impact of the COVID-19 pandemic on the demand for antibiotics and access to antibiotics varied among the respondents based on their experiences. A total of 32% of the respondents reported a decrease in their need for antibiotics due to a lower incidence of illness. Conversely, only 8% experienced an increase in their antibiotic needs. In terms of access to antibiotics, 37% of the respondents stated that their access remained unchanged. However, 10% of the respondents reported experiencing limited access to antibiotics, which was attributed to difficulties in obtaining prescriptions or accessing pharmacies (Figure 12).

### Implications for policy

**Figure 13:**
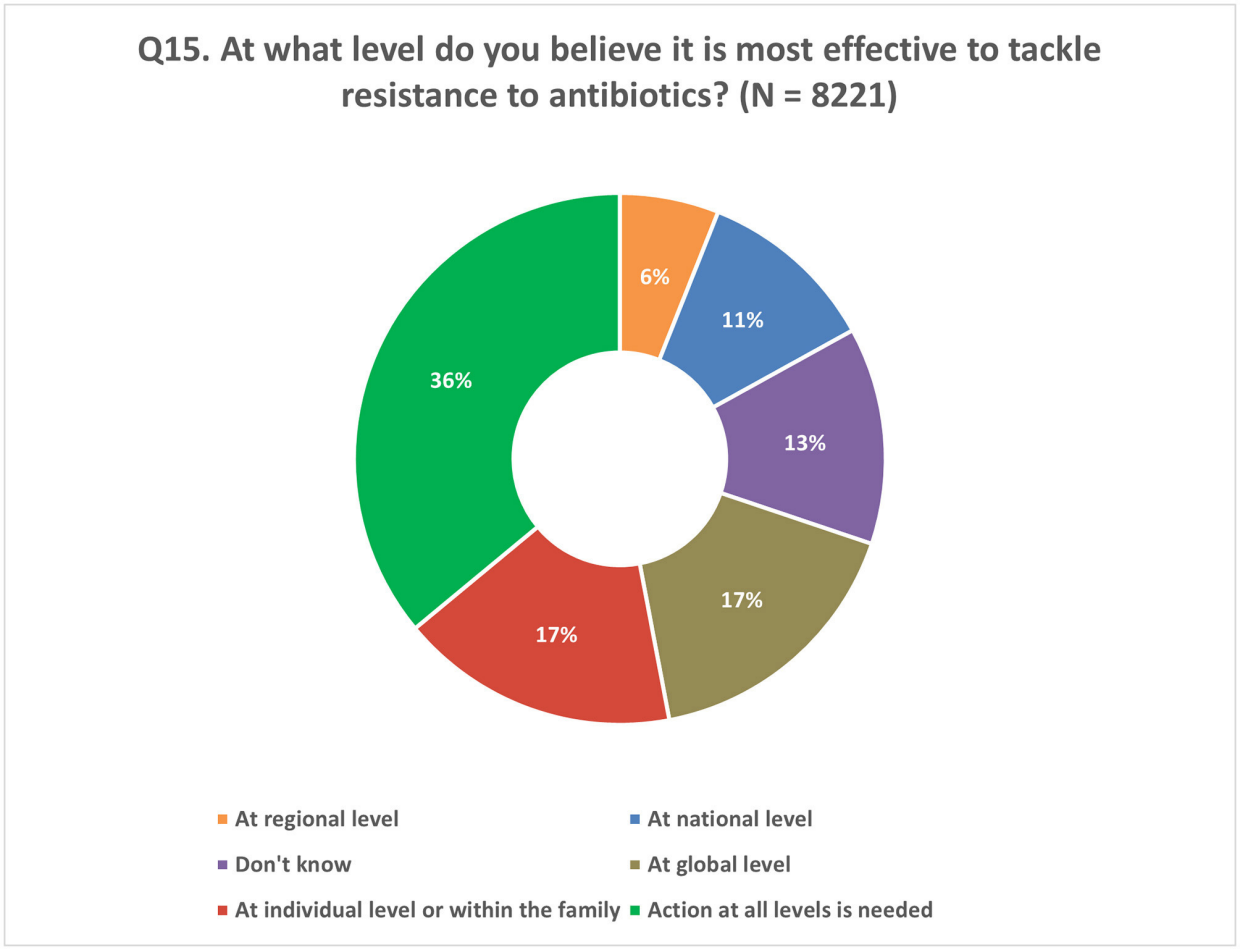
Respondents' understanding of the correct level of policy intervention to tackle AMR. Respondents were allowed to provide only one response for Q15.

In response to the question about the most effective level to address antibiotic resistance, 36% of the respondents emphasized the importance of taking actions at all levels, indicating that a comprehensive approach involving various stakeholders is crucial for tackling antibiotic resistance. On the other hand, 17% of the respondents believed that actions taken by individuals or within the family unit hold the most effectiveness in combating AMR. Additionally, 34% of the respondents expressed the opinion that addressing AMR would be most effective at the global, regional, or national level (Figure 13).

## Discussion

Addressing the irrational use of antibiotics is an important aspect in the fight against AMR. Most of the inappropriate antibiotic use in the human sector occurs at the intersection of the health-care system and the general public, emphasizing the need for interventions at this interface. The survey sought to understand various aspects of appropriate antibiotic use and AMR among the general population. It also examined the impact of awareness raising campaigns and other sources of antibiotic and AMR-related information. The results of the present study shed light on the knowledge gaps that exist among individuals surveyed regarding appropriate antibiotic use within the community.

This study highlights that half of the participants reported having taken antibiotics in the last year, and over half of those respondents received their antibiotics through a medical prescription. This also means that roughly half of individuals who took antibiotics did not have a prescription in spite of the legislation in place prohibiting sales of antibiotics without prescription in all the surveyed countries (26). According to a systematic review published in 2019, the global prevalence of non-prescription antibiotic sales was estimated to be 62% (12). A recent study conducted in community pharmacies across eight selected Member States in the Region revealed variations in the utilization of prescriptions for antibiotic supply, ranging from 23 to 97% (13). This highlights the issue of OTC sales of antibiotics and the need for stricter enforcement of existing laws regulating prescription medications while also ensuring access to them.

There may also be some confusion among participants as to what constitutes a formal medical prescription, as 14% of respondents stated they took antibiotics “administered by a medical practitioner” and 20% stated they took antibiotics without a prescription from a pharmacy. The survey did not explore the participants' perceptions regarding the definition of a medical practitioner. These perceptions may be particularly convoluted in contexts where existing laws prohibiting the sale of antibiotics OTC may not be rigorously enforced. As such, it is important to address these misconceptions and ensure a clear understanding of the appropriate channels for obtaining antibiotics.

Furthermore, financial barriers and promoting equitable access to health-care services are crucial in ensuring appropriate antibiotic use and mitigating the risks associated with AMR. In our survey, nearly 60% of respondents reported difficulty in paying bills (either sometime or frequently; see Table 1). Considering the influence of socioeconomic status on health, future surveys in the Region should look into how such factors might influence antibiotic consumption.

The survey further sought to elucidate participants' reasons for using antibiotics, with a majority indicating suspected viral infections. Responses from Q3 were reorganized into groups based on the suspected likelihood for antibiotic need. Without more detailed follow-up questions or clinical information, it is not possible to be certain whether antibiotics were used for the correct indication. However, there is a clear grouping of potential viral, bacterial, and unknown categories (27–30). The majority of respondents stated that they used antibiotics for what could be considered suspected viral infections (e.g., cold, sore throat, flu). This trend of unjustified utilization is also observed in the 2022 Eurobarometer survey, albeit at lower levels, where a large proportion of respondents from the EU/EEA Member States cite reasons for taking antibiotics that are either unjustified (i.e., probable viral infections or symptoms such as fever. For example, sore throat – 13%; cold – 11%; flu – 10%; and fever – 10%) or questionable (such as bronchitis – 13% or pneumonia – 4%) as they may be either viral or bacterial, requiring confirmatory testing (24).

This reflects findings from later in the survey, where almost half of all respondents incorrectly indicated that antibiotics are effective against viruses and colds. Findings from the 2022 Eurobarometer survey similarly show that only about 50% of participants know that antibiotics are ineffective against viruses. In fact, 84% of respondents in our survey could not correctly validate all four statements in Q5.1 to Q5.4 (see Supplementary File 1). However, this set of questions also identifies partial knowledge and awareness that could arise from personal experience. This could also indicate that people might be taking antibiotics for incorrect indications without realizing it. This information illustrates gaps in knowledge and identifies rationale for which the general population seeks antibiotics. It further suggests that there is room for more tailored, and perhaps seasonal, communication campaigns at the population level to increase awareness about appropriate antibiotic use. Meanwhile, it should be also noted that gaps in knowledge are not the only drivers of irrational antibiotic use when rigorous health systems are not in place to strictly control access to antibiotics with prescription as discussed earlier.

The self-reported consumption of antibiotics in this survey was 50% compared to 23% in the EU/EEA Member States in the 2022 Eurobarometer survey (the lowest reported levels since 2009) (24). The burden of appropriate antibiotic use must not only be placed on consumers, but must be shared between health-care professionals, governments, and health systems, to name a few.

In relation to this, it is important to assess the public's recollection of receiving information on the correct use of antibiotics. Only a small subset of respondents across all Member States (range: 23–48%) confirmed receiving any information about antibiotics use, leaving much of the population yet to be reached by awareness campaigns. This lack of information could be one of the possible explanations of why there is such a discrepancy in knowledge about the effectiveness of antibiotics against colds. As such, this could correlate with irrational use of antibiotics, and thereby contribute to AMR (31, 32).

Regarding respondents' understanding of adherence to an antibiotic treatment regimen, 72% of respondents correctly stated that they should follow the full course of treatment as prescribed by their doctor. A similarly high number of participants (85%) in the 2022 Eurobarometer survey responded correctly to the same question (24). WHO's recommendations are to use evidence-based prescribing and adhering to the dosage and duration of a treatment regimen, as prescribed by a licensed clinician. This also means that feeling better, or an improvement in symptoms, does not always mean that an infection has cleared (33). By cutting short a prescribed antibiotic treatment course, a patient is at risk of having to restart the treatment for a possibly persistent infection, or possibly require a stronger antibiotic if resistance develops (6).

Participants were also asked about their sources of information on antibiotics. The study indicates trust is placed in health-care professionals, particularly doctors, highlighting that health-care professionals are currently the most prevalent source of conveying information to the public. It is worth noting that, besides professional or health-care facilities, roughly one-third of respondents stated that they received information about unnecessary use of antibiotics from the internet or social media, and about one-quarter stated they receive information from family or friends. This means that there are multiple entry points for awareness campaigns and health literacy programs to target beyond formal health-care settings and to dispel misinformation. Increasing awareness in certain population groups could also possibly have a snowballing effect through disseminating correct information among their family and friend networks in person, but also through social media networks and through online platforms. Awareness raising campaigns, therefore, could be tailored to channeling information through the above three main target groups and platforms.

It is also important to note that almost 40% of respondents showed interest in gaining knowledge about the appropriate usage of antibiotics and their purpose. Among EU/EEA Member States surveyed in the 2022 Eurobarometer survey, even more respondents stated they were interested in receiving additional information about antibiotics (79%) (24). Additionally, over 30% of respondents expressed their interest in acquiring information about AMR. A majority of respondents (78%) considered doctors as the most reliable source of information on antibiotics. This ties back to the question on source of information (Q8), where most respondents stated that they received health-related information, including about antibiotics, from health-care professionals and facilities.

The aforementioned findings underscore the significance of enhancing communication between the patient and the physician during the prescription of antibiotics. It is essential for doctors to explain, in an easily understandable manner, the reasons behind the prescription of antibiotics and provide detailed instructions on how to take them. Although the Eurobarometer survey showed similar results on the trustworthy information source on antibiotics, a notable difference was observed in the trust in pharmacists (40% in Eurobarometer vs. 29% in this survey). Despite being the primary provider of antibiotics to patients, pharmacists were not as highly trusted by the respondents. Given that antimicrobials are still commonly sold without a prescription in many of the surveyed Member States, despite the existence of laws prohibiting this practice (12), emphasis should be made on the crucial role of pharmacists in promoting responsible antibiotic use.

This questionnaire was conducted during the COVID-19 pandemic, and it retrospectively surveyed respondents about their experiences and perceptions of antibiotics over the previous year. The impact of the pandemic on the respondents' demand for antibiotics was found to vary in terms of their experiences. Specifically, about one-third of the respondents reported a decrease in their need for antibiotics owing to a reduced incidence of illness. It is noteworthy that the Eurobarometer survey reported a higher proportion (45%) of respondents who experienced a similar reduction in their demand for antibiotics. This highlights the need to closely monitor and address changes in antibiotic usage patterns during public health crises.

Overall, participants agreed that to effectively combat the issue of antibiotic resistance, a multi-level approach is necessary, encompassing efforts at the individual, national, regional and global levels. This includes promoting responsible antibiotic use through education, awareness campaigns, policy enforcement, and collaboration among health-care professionals, policymakers, and the public. By addressing the gaps in KAB related to antibiotic use, we can contribute to the global fight against AMR and ensure the continued effectiveness of antibiotics for future generations.

Our study is subject to several limitations that should be acknowledged. Firstly, the sampling approach focused solely on capital cities and though survey sites within each city were randomly selected from a predefined sampling frame and participants were identified using systematic random sampling, selection bias cannot be ruled out. For example, data collection at a particular time of day at a given site might influence the type of participants. This limits, the representativeness and generalizability of our findings to rural areas and other regions within the Member States, and comparison with the results of the Eurobarometer survey challenging. Secondly, one of the survey questions (Q10) presented only positive response choices, potentially leading respondents to answer in a socially desirable manner, thereby influencing the accuracy of their responses. Thirdly, recall bias may have influenced participants' ability to accurately remember and report events that occurred over a 12-month period. Fourthly, the potential for interviewer bias exists, given that the original questionnaire was developed for EU/EEA settings and was now used for the first time outside of that context in diverse settings. Although efforts were made to mitigate this bias through standardized training and pilot sessions, variations in interviewer techniques and interpretations may still have influenced participant responses. Lastly, social desirability bias may have impacted participants' responses, as individuals tend to provide answers they perceive as socially or morally acceptable, potentially leading to an overestimation of positive behaviors and an underestimation of negative behaviors. Recognizing these limitations, it is important to interpret our findings in light of these inherent constraints.

## Conclusion

To our knowledge, this is the first cross-national survey evaluating and highlighting the gaps in KAB concerning antibiotic use and AMR in these 14 Member States of the WHO European Region. These findings emphasize the urgent need for targeted awareness campaigns and educational initiatives aimed at bridging these gaps as well as providing baseline information for future evaluations. By proactively addressing these challenges, we can foster a culture of responsible antibiotic use and make major strides in our global efforts to combat the threat of AMR.

## Data availability statement

The datasets presented in this article are not readily available because all the data and materials are owned by the ministries of health of the participating Member States, WHO Regional Office for Europe and participating country offices. Requests to access the datasets should be directed to WHO Regional Office for Europe.

## Ethics statement

The study was exempted from review of the WHO Ethics Review Committee (Protocol Number ERC.0003790). Participants were informed about the objective, outcomes and any associated risks of the study and were only included for interviews if they provided consent.

## Author contributions

SS-P: Data curation, Formal analysis, Investigation, Methodology, Software, Supervision, Validation, Visualization, Writing—original draft, Writing—review & editing. PA: Investigation, Validation, Writing—original draft, Writing—review & editing. DW: Conceptualization, Investigation, Resources, Validation, Writing—review & editing. KI: Conceptualization, Investigation, Methodology, Supervision, Validation, Writing—original draft, Writing—review & editing. KK: Conceptualization, Funding acquisition, Investigation, Project administration, Resources, Supervision, Writing—original draft, Writing—review & editing.
