# Supplementary material for: Knowledge, attitudes and behaviors on antimicrobial resistance among general public across 14 member states in the WHO European region: results from a cross-sectional survey
Source: Front Public Health. 2023 Nov 23;11:1274818. doi: 10.3389/fpubh.2023.1274818 (PMC10704021; doi:10.3389/fpubh.2023.1274818)
Supplement: Supplementary file 1 [file Table_1.DOCX]

# Supplement 1 Questionnaire

**Sociodemographic information**

1. What is your gender? (Female, Male, other, do not wish to answer)
2. What is your age group? (18-24, 25-39, 40-54, 55+)
3. How old were you when you finished your education? (<15, 16-19, 20+, still studying)
4. What is your socio-professional category? (self-employed, managers, other white office worker, manual workers, house persons, unemployed, retired, students)
5. Difficulties paying bills (most of the time, from time to time, almost never/Never)

**Q1 Have you taken any antibiotics orally such as tablets, powder or syrup in the last 12**

**months?** *(ONE ANSWER ONLY)*

- Yes 1,
- No 2,
- Refusal 3,
- Don’t Know 4

***ASK Q2, Q3 AND Q4 IF “YES”, CODE 1 IN Q1 – OTHERS GO TO Q5***

**Q2 How did you obtain the last course of antibiotics that you used?**

*(SHOW SCREEN – READ OUT – ROTATE - ONE ANSWER ONLY)*

- *From a medical prescription* 1,
- Administered by a medical practitioner 2,
- You had some left over from a previous course 3,
- Without prescription from a pharmacy 4,
- Without prescription from elsewhere 5,
- Don’t remember 6,
- Refusal 7,
- Don’t Know 8

**Q3 What was the reason for last taking the antibiotics that you used?**

*(SHOW SCREEN – READ OUT – ROTATE - MULTIPLE ANSWERS POSSIBLE)*

- Pneumonia (an infection causing an inflammation of one or both lungs) 1,
- Bronchitis (inflammation and swelling of the bronchi, the airways that carry airflow from

the trachea into the lungs) 2,

- Rhino pharyngitis (inflammation of the mucous membrane of the nose and pharynx) 3,
- Flu 4,
- Cold 5,
- Sore throat 6,
- Cough 7,
- Fever 8,
- Headache 9,
- Diarrhea 10,
- Urinary tract infection 11,
- Skin or wound infection 12,
- Other 13,
- Do not wish to answer 14,
- Don’t Know 15,
- Covid-19 16

**Q4 Did you have a test, for example a blood or urine test, or throat swab, to find out what**

**was causing your illness, before or at the same time as you started antibiotics ?**

*(ONE ANSWER ONLY)*

- Yes 1,
- No 2,
- Don’t remember 3,
- Do not wish to answer 4,
- Don’t Know 5

***ASK ALL***

**Q5 For each of the following statements, please tell me whether you think it is true or**

**false.**

*(SHOW SCREEN – READ OUT – ONE ANSWER PER LINE)*

1-True, 2-False, 3- Don’t Know

- 1 Antibiotics kill viruses 1 2 3
- 2 Antibiotics are effective against colds 1 2 3
- 3 Unnecessary use of antibiotics makes them become ineffective 1 2 3
- 4 Taking antibiotics often has side-effects such as diarrhea 1 2 3

**Q6 When do you think you should stop taking antibiotics once you have begun a course of**

**treatment?**

*(READ OUT – ONE ANSWER ONLY)*

- When you feel better 1,
- When you have taken all of the antibiotics as directed by your doctor 2,
- Other 3,
- Don’t Know 4

**Q7 In the last 12 months, do you remember getting any information about not taking**

**antibiotics unnecessarily, for example for a cold?**

*(ONE ANSWER ONLY)*

- Yes 1
- No 2
- Don’t Know 3

***ASK Q8 AND Q9 IF “YES”, CODE 1 IN Q7 – OTHERS GO TO Q11***

**Q8 Where did you get this information about not taking antibiotics unnecessarily?**

*(SHOW SCREEN – READ OUT – MULTIPLE ANSWERS POSSIBLE)*

- From a doctor 1,
- From a pharmacist 2,
- From another health professional (e.g. nurse) 3,
- From a family member or friend 4,
- From a TV advertisement 5,
- On the Internet or in online social networks 6,
- In a leaflet or on a poster 7,
- In a newspaper 8,
- On the TV news or other programmes 9,
- On the radio 10,
- Other 11,
- Don’t Know 12

**Q9 Did the information that you received change your views on using antibiotics?**

*(ONE ANSWER ONLY)*

- Yes 1,
- No 2,
- Don’t Know 3

***ASK Q10 IF “YES”, CODE 1 IN Q9 – OTHERS GO TO Q11***

**Q10 On the basis of the information you received, how do you now plan to use antibiotics?**

*(SHOW SCREEN – READ OUT – ROTATE - MULTIPLE ANSWERS POSSIBLE)*

- You will always consult a doctor when you think you need antibiotics 1,
- You will no longer self-medicate with antibiotics 2,
- You will no longer take antibiotics without a prescription from a doctor 3,
- You will no longer keep left over antibiotics for next time you are ill 4,
- You will give left-over antibiotics to your relatives or friends when they are ill 5,
- Other 6,
- None 7,
- Do not wish to answer 8,
- Don’t know 9

***ASK ALL***

**READ OUT: Antimicrobial Resistance is the ability of micro-organisms to resist antimicrobial**

**treatments, especially antibiotics.**

**Q11 On which topics, if any, would you like to receive more information?**

*(SHOW SCREEN – READ OUT – ROTATE - MULTIPLE ANSWERS POSSIBLE)*

- Resistance to antibiotics 1,
- How to use antibiotics 2,
- Medical conditions for which antibiotics are used 3,
- Prescription of antibiotics 4,
- Links between the health of humans, animals and the environment 5,
- Other 6,
- None 7,
- I don’t want to receive more information on these issues 8,
- Don’t know 9

**Q12 Which of the following sources of information would you use in order to get**

**trustworthy information on antibiotics?**

*(SHOW SCREEN – READ OUT – MAX. 3 ANSWERS)*

- A doctor 1,
- A nurse 2,
- A pharmacy 3,
- A hospital 4,
- Another health care facility 5,
- Family or friends 6,
- An official health-related website (e.g. a website set up by the national government/ public
- health body/ World Health Organization)7,
- A health-related personal blog 8,
- Another health-related website 9,
- Online social networks 10,
- TV 11,
- Newspapers or magazines 12,
- The radio 13,
- Other 14,
- You are not interested in finding information on antibiotics
- 15
- Don’t know 6

**Q13: If you suffered from COVID-19, did you take any antibiotics? [MULTIPLE ANSWERS POSSIBLE]**

- I did not get COVID-19 1,
- No, I did not take antibiotics 2,
- Yes, from a medical prescription 3,
- Yes, administered by a medical practitioner (e.g. in hospital, in a clinic) 4,
- Yes, I had some left over from a previous course 5,
- Yes, without prescription from a pharmacy 6,
- Yes, without prescription from elsewhere 7,
- Don’t remember (spontaneous) 8,
- Refusal (spontaneous) 9,
- Don’t know 10

**Q14: What kind(s) of impact did the COVID-19 pandemic have (e.g. due to restrictions, new personal protective measures such as hand hygiene, masks and physical distancing) on your need to take antibiotics? [MULTIPLE ANSWERS POSSIBLE]**

- It decreased my need to take antibiotics because I fell ill less often due to strengthened personal protective measures 1,
- It decreased my need for antibiotics because I fell ill less often during the containment period 2,
- It restricted my access to antibiotics as I couldn’t go to the doctor to get a prescription 3,
- It restricted my access to antibiotics as I couldn’t go to the pharmacy 4,
- It increased my need for antibiotics 5,
- My access to antibiotics remained the same 6,
- Don’t remember 7,
- Refusal 8,
- Don’t know 9

**Q15 At what level do you believe it is most effective to tackle resistance to antibiotics?**

*(READ OUT – ONE ANSWER ONLY)*

- At individual level or within the family 1,
- At regional level 2,
- At national level 3,
- At global level 4,
- Action at all levels is needed 5,
- Don’t know 6

**READ OUT: Now, let’s talk about the use and effects of antibiotics in farm animals, i.e. animals**

**used for consumption (meat, dairy products, etc.).**

**Q16 To what extent do you agree or disagree that sick farm animals should be treated**

**with antibiotics if this is the most appropriate treatment? – this question was not part of analysis**

*(ONE ANSWER ONLY)*

- Totally agree 1,
- Tend to agree 2,
- Tend to disagree 3,
- Totally disagree 4,
- Don’t know 5

Text for Informed Consent

Hello, my name is ……….. I am from ……………...

We are conducting a research survey on antibiotic use and antimicrobial resistance on behalf of researchers at ……………………...

We are not asking for money or selling anything. We are looking for people who are more than 18 years and could answer 16 questions. All survey participants are being approached randomly for participation, and the survey takes about 15 minutes to complete.

Do you have a few minutes to do our survey, and are you more than 18 years old?

- Yes, continue
- No – no time or less than 18 years old

Now, I am going to read you some important information about the survey.

This is a research project on antibiotic use and antimicrobial resistance. The research aims at understanding knowledge, attitudes and behavior of people around antibiotics and antimicrobial resistance. We are going to ask you 16 questions regarding your knowledge, experiences and views around antibiotics and antimicrobial resistance.

Identification information will not be asked or recorded. Your participation is voluntary and you may refuse to answer any questions you do not wish to answer. You have the right to withdraw at any time, without penalty.

The data collected from this research will be summarized in reports and used for antimicrobial awareness campaign in [COUNTRY] by World Health Organization. No individual person will be knowingly identifiable from the summarized results.

This study has been reviewed and received ethics clearance through “the World Health Organization Ethics Review Committee”. Should you have any comments or concerns resulting from your participation in this study, I can provide the contact information for the ………………________________.

Are you ready to continue?

- Yes, go to begin survey
- No, go to better time

I will begin the survey now.
