# Supplementary material for: Knowledge, attitudes and behaviors on antimicrobial resistance among general public across 14 member states in the WHO European region: results from a cross-sectional survey
Source: Front Public Health. 2023 Nov 23;11:1274818. doi: 10.3389/fpubh.2023.1274818 (PMC10704021; doi:10.3389/fpubh.2023.1274818)
Supplement: Supplementary file 2 [file Table_2.DOCX]

**Supplementary File 2**

**Table 1. List of participating countries in the survey**

| **Country** | **Survey cities** | **Country code** |
| --- | --- | --- |
| Albania | Tirana | ALB |
| Armenia | Yerevan | ARM |
| Azerbaijan | Baku | AZE |
| Belarus | Minsk | BLR |
| Bosnia and Herzegovina* | Mostar; Banja Luka | BIH |
| Georgia | Tbilisi | GEO |
| Kazakhstan | Almaty | KAZ |
| Kyrgyzstan | Bishkek | KGZ |
| Montenegro | Podgorica | MNE |
| North Macedonia | Skopje | MKD |
| Republic of Moldova | Chișinău | MDA |
| Tajikistan | Dushanbe | TJK |
| Türkiye | Ankara | TUR |
| Uzbekistan | Tashkent | UZB |

^*^For BIH, the survey took place in two cities – Mostar and Banja Luka.

**Table 2. Total number of interviews conducted across countries that met eligibility criteria and provided consent**

| **Country Code** | **Interviews** |
| --- | --- |
| ALB | 525 |
| ARM | 570 |
| AZE | 514 |
| BIH^*^ | 1055 |
| BLR | 548 |
| GEO | 548 |
| KAZ | 553 |
| KGZ | 528 |
| MDA | 572 |
| MKD | 556 |
| MNE | 531 |
| TJK | 627 |
| TUR | 545 |
| UZB | 549 |
| **Grand Total** | **8221** |

^*^ The total number of surveys in BIH for the two cities was 1055.


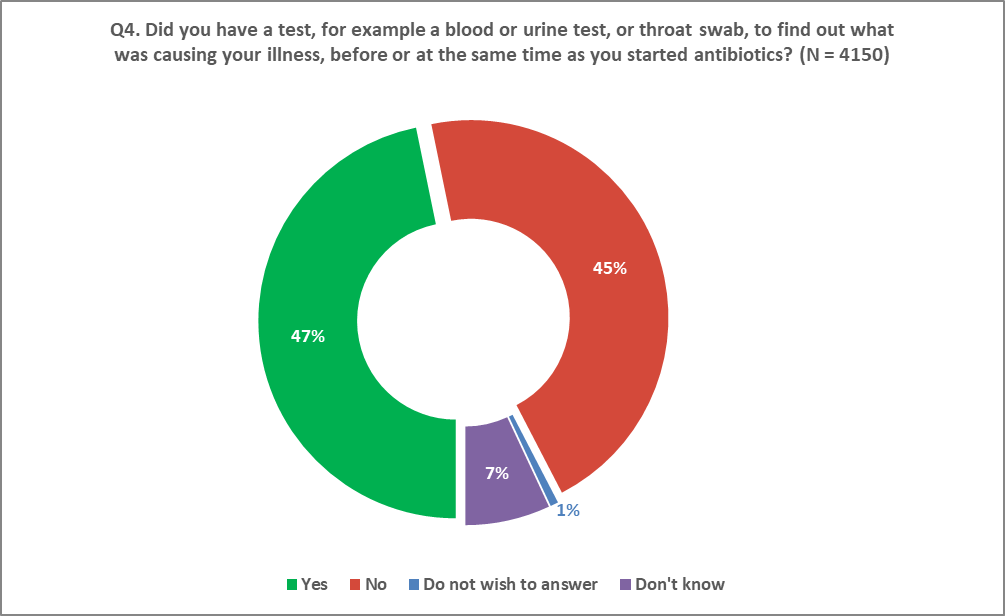


**Fig. 1a. Diagnosis of the cause of illness before or at the time of initiating antibiotics**


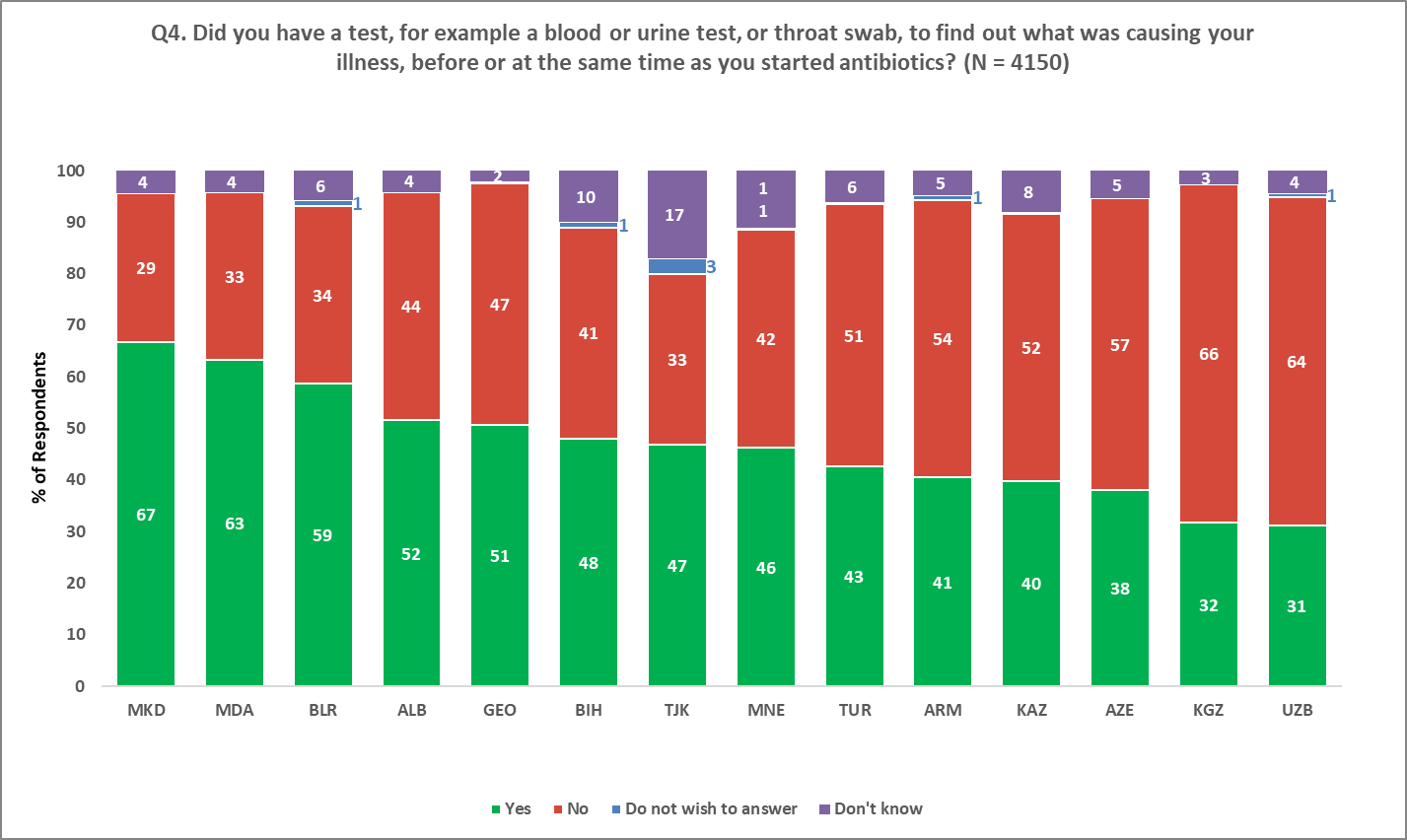


**Fig. 1b. Diagnosis of the cause of illness before or at the time of initiating antibiotics at national level**

Note: Respondents were allowed to provide only one response for Q4.


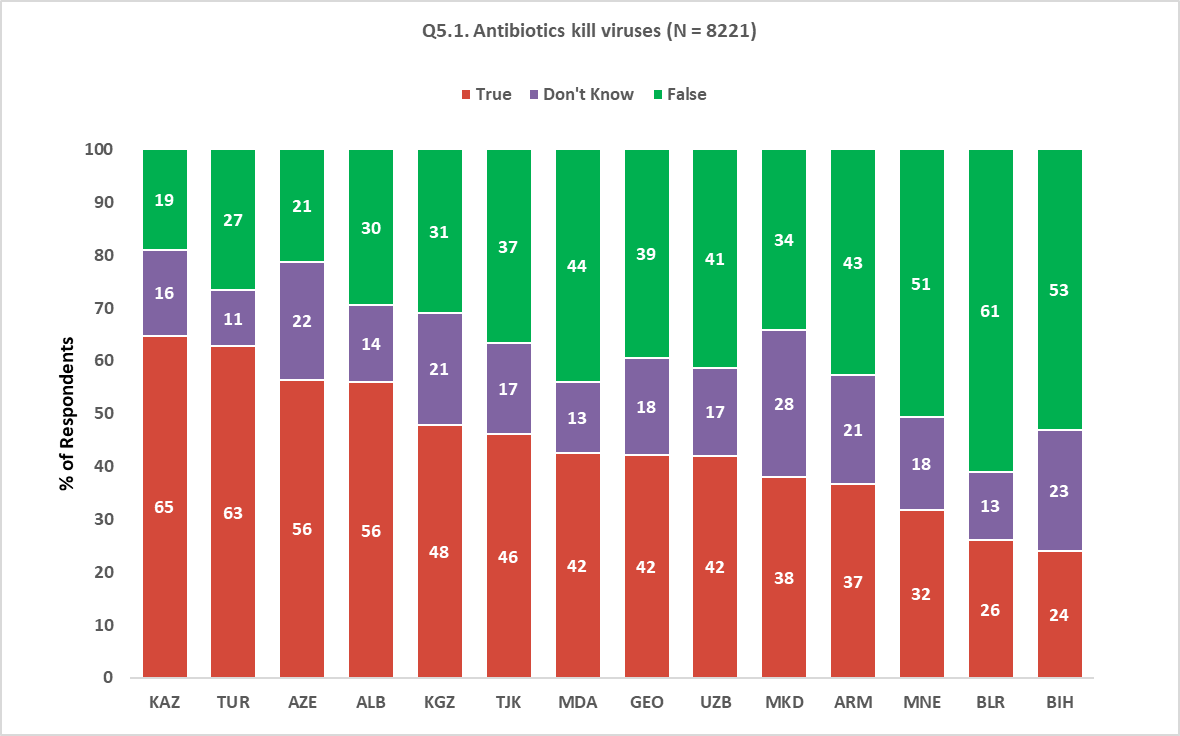


**Fig. 2a. Do antibiotics kill viruses?**


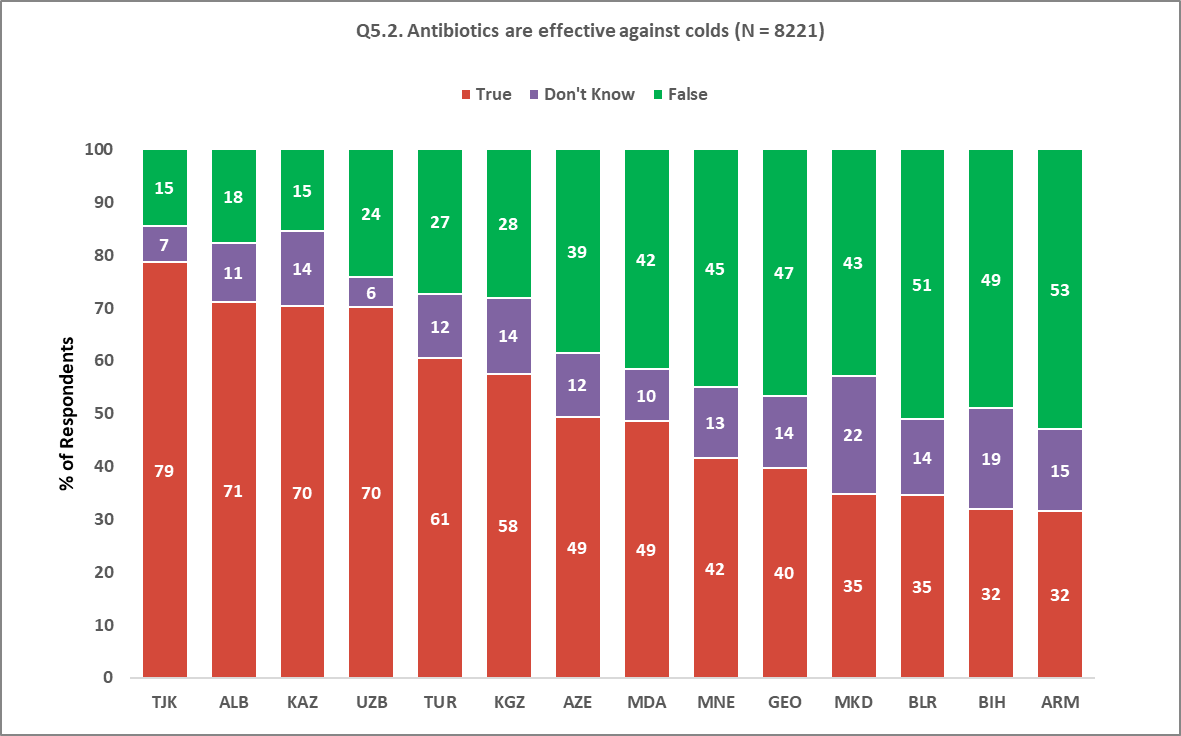


**Fig. 2b. Are antibiotics effective against colds?**


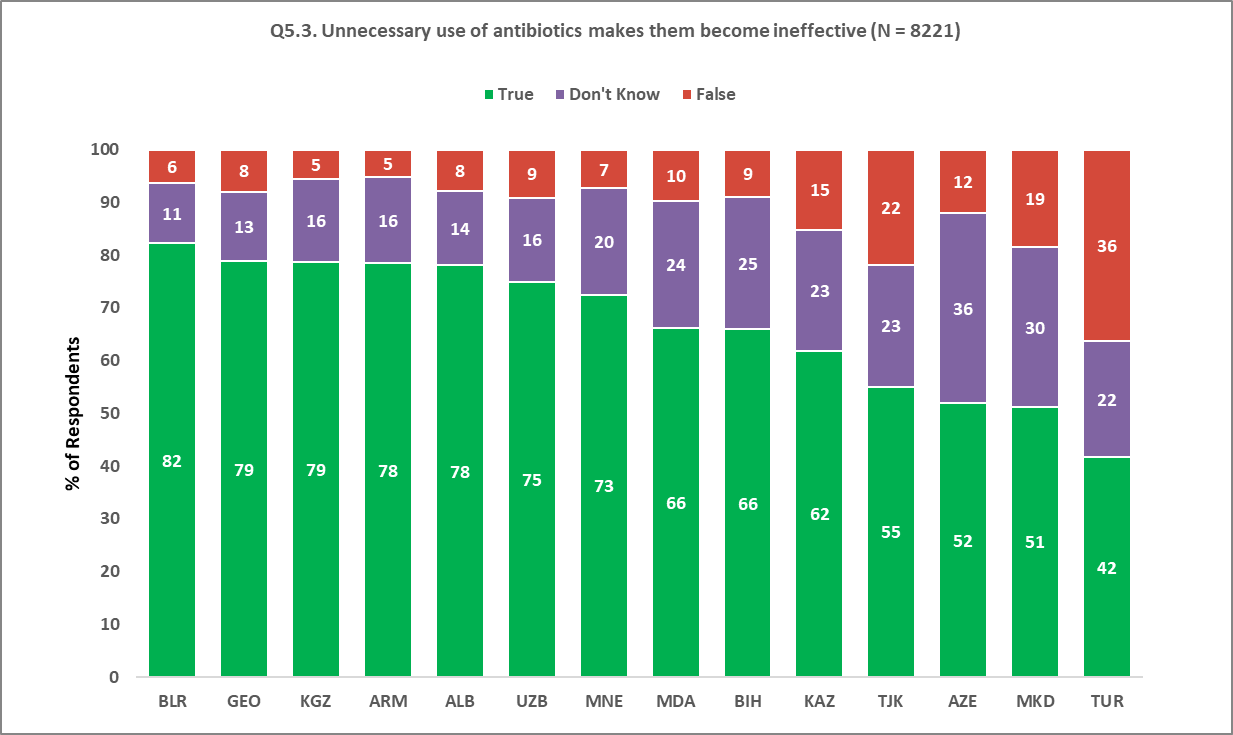


**Fig. 2c. Does unnecessary use of antibiotics make them ineffective?**


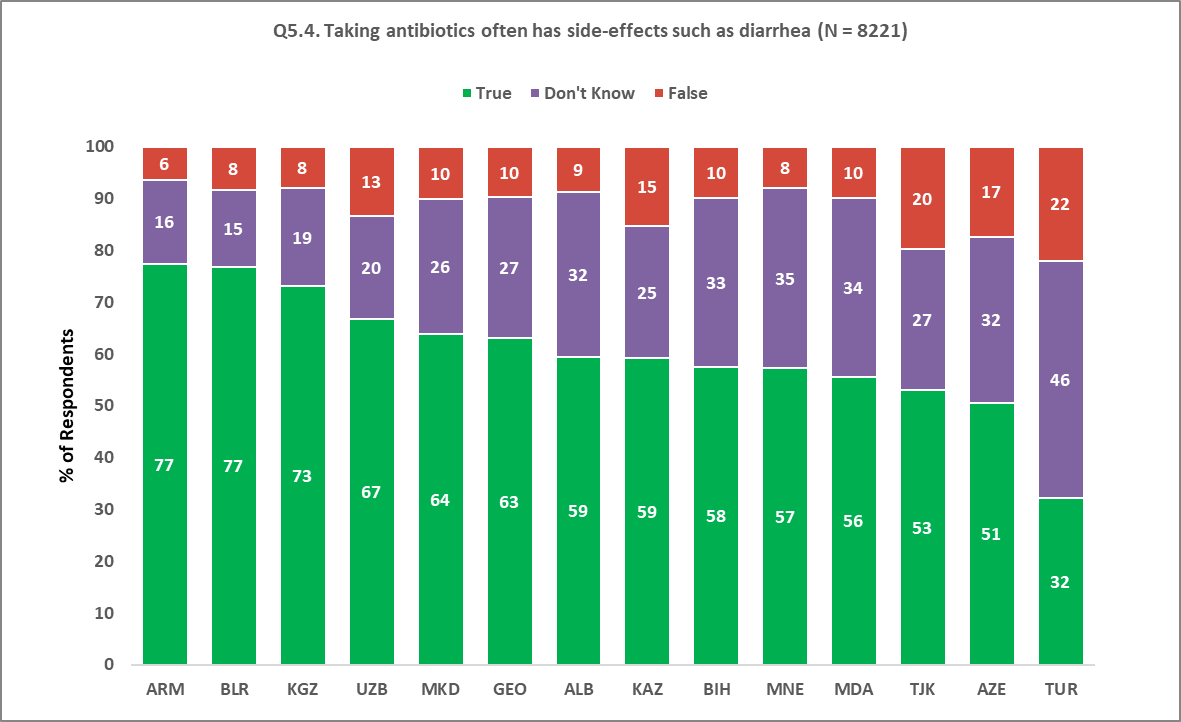


**Fig. 2d. Does taking antibiotics often have side-effects such as diarrhoea?**

Note: Respondents were allowed to provide only one response for Q5.1 to Q5.4.

**
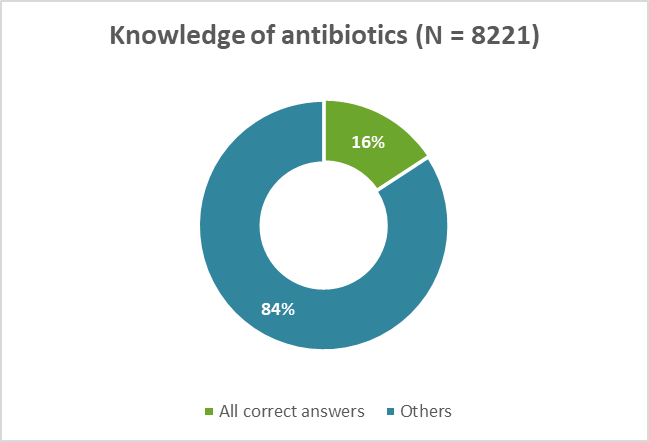
**

**Fig. 2e. Percentage of participants who correctly validated all of the questions (Q5.1 to Q5.4) on antibiotics knowledge**

**
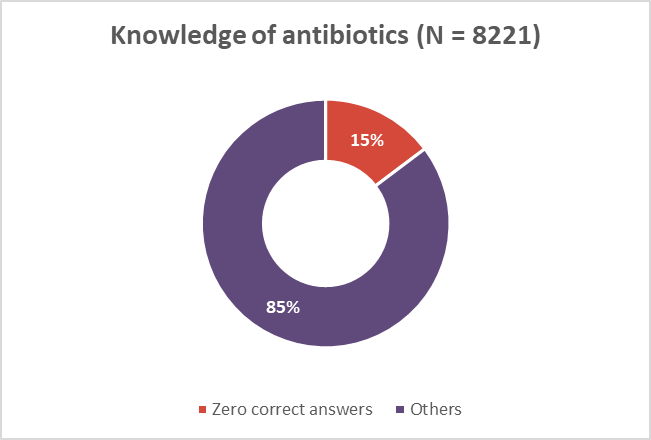
**

**Fig. 2f. Percentage of participants who could not validate any of the questions (Q5.1 to Q5.4) on antibiotics knowledge**


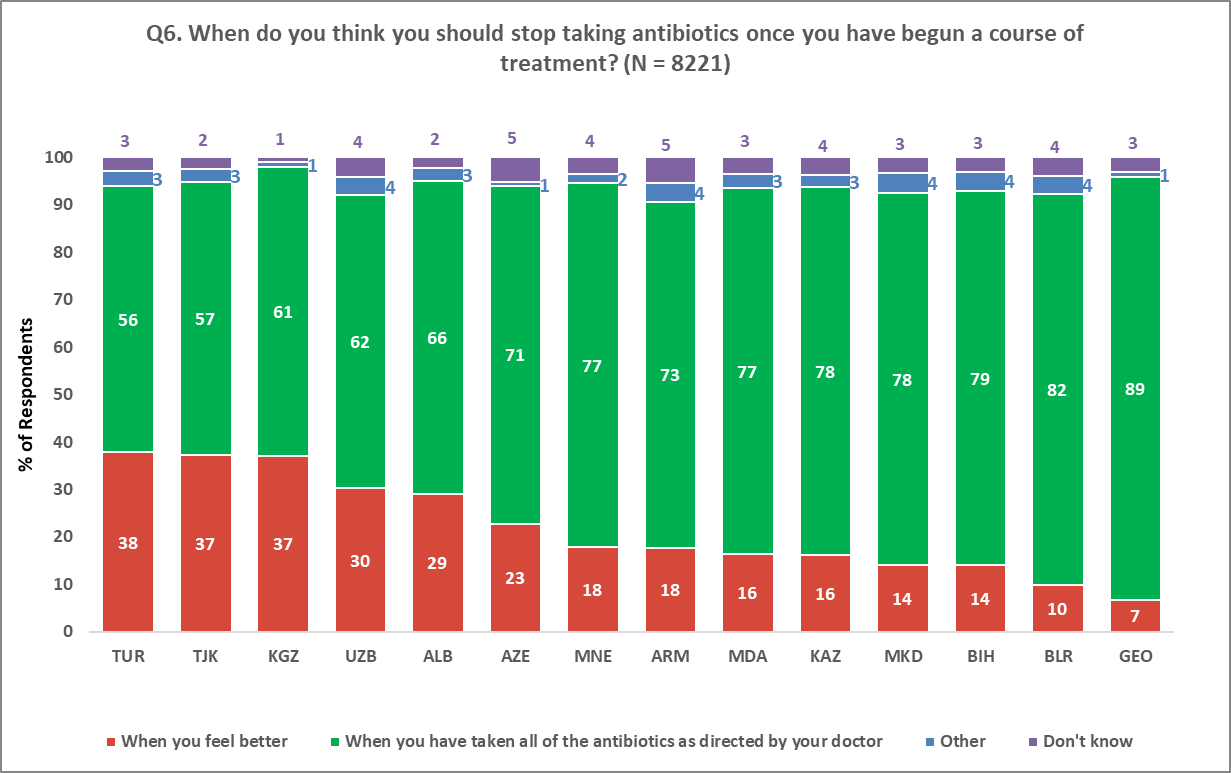


**Fig. 3. Compliance to recommended treatment at national level**

Note: Respondents were allowed to provide only one response for Q6.


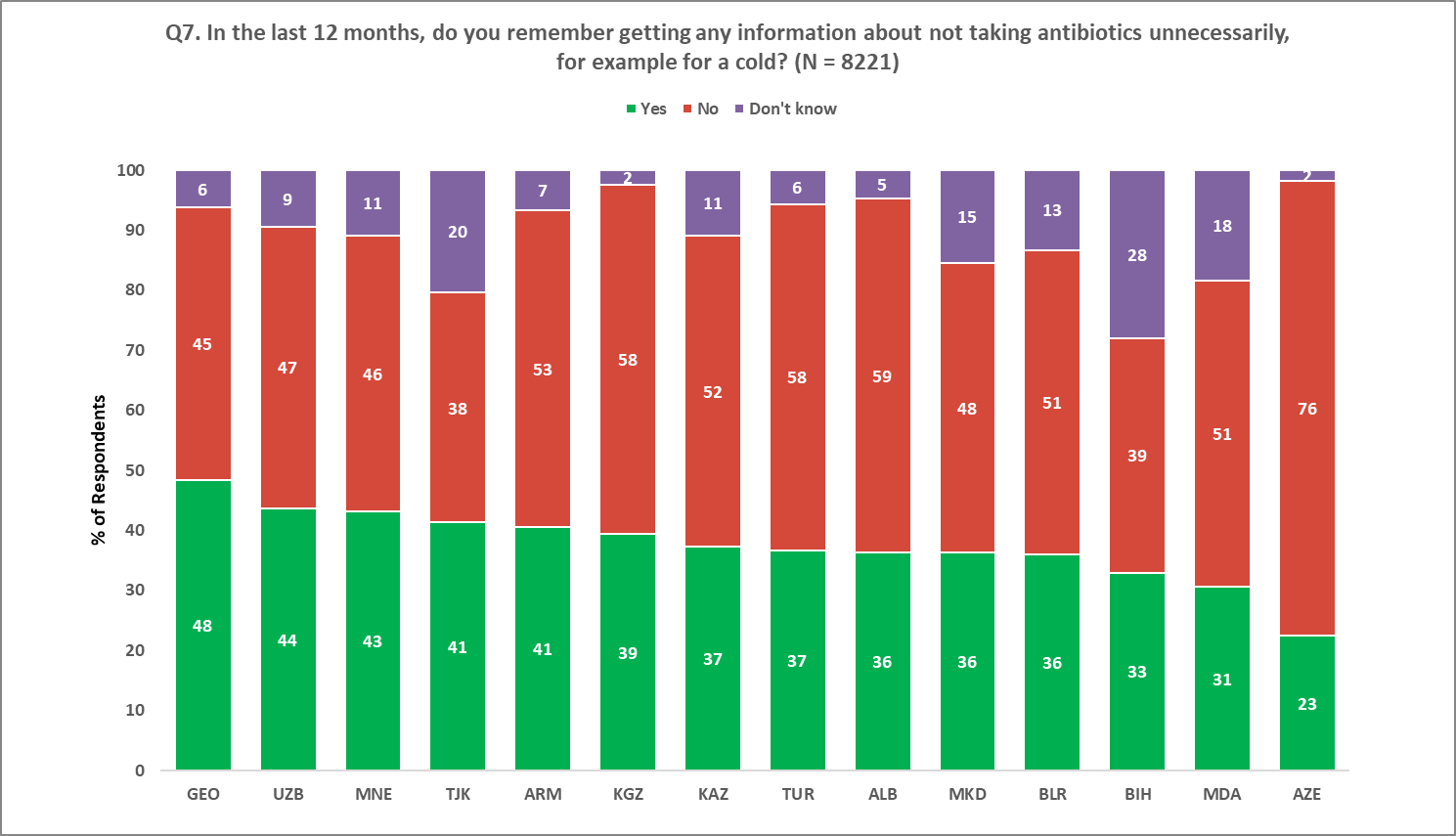


**Fig. 4. Information received by participants on unnecessary use of antibiotics in the last 12 months at national level**

Note: Respondents were allowed to provide only one response for Q7.


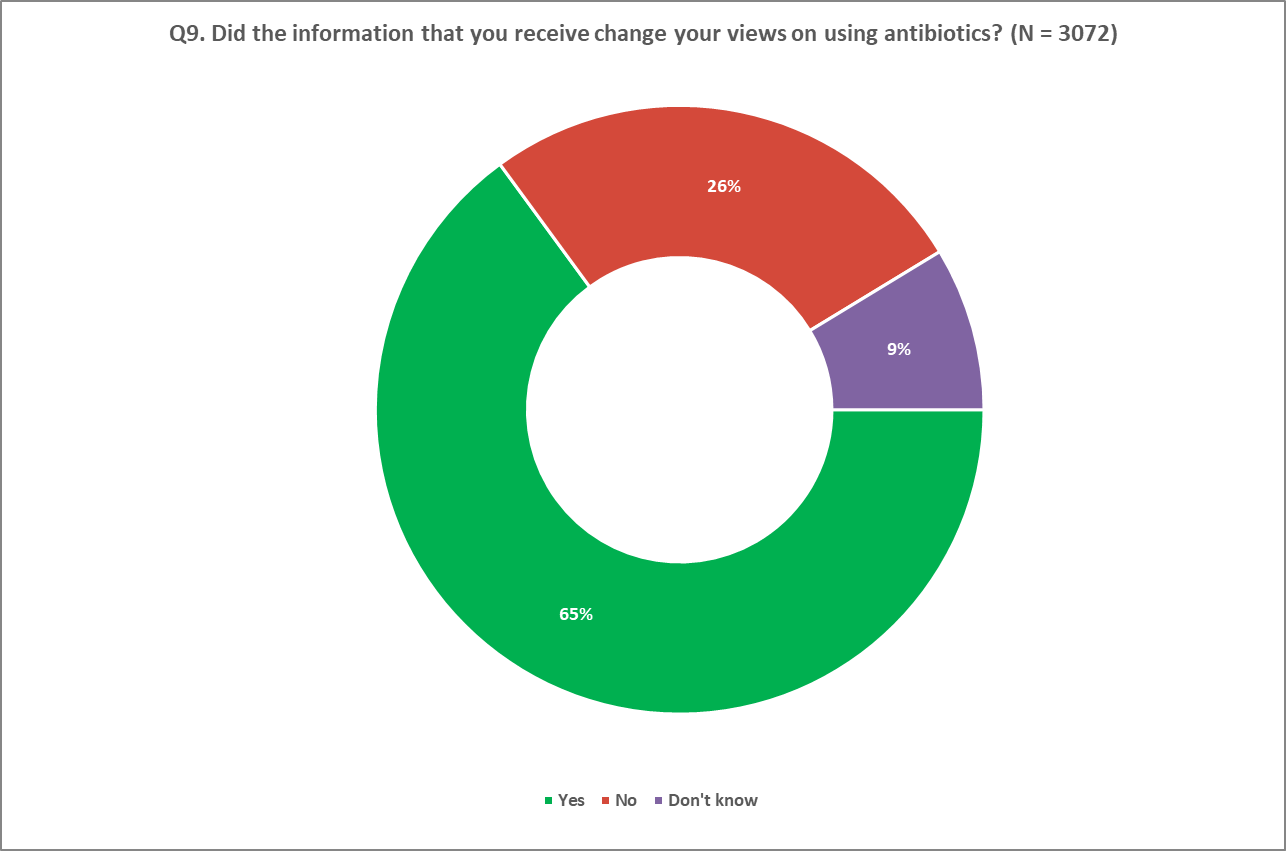


**Fig. 5a. Information leading to participants’ behaviour change concerning antibiotic use**


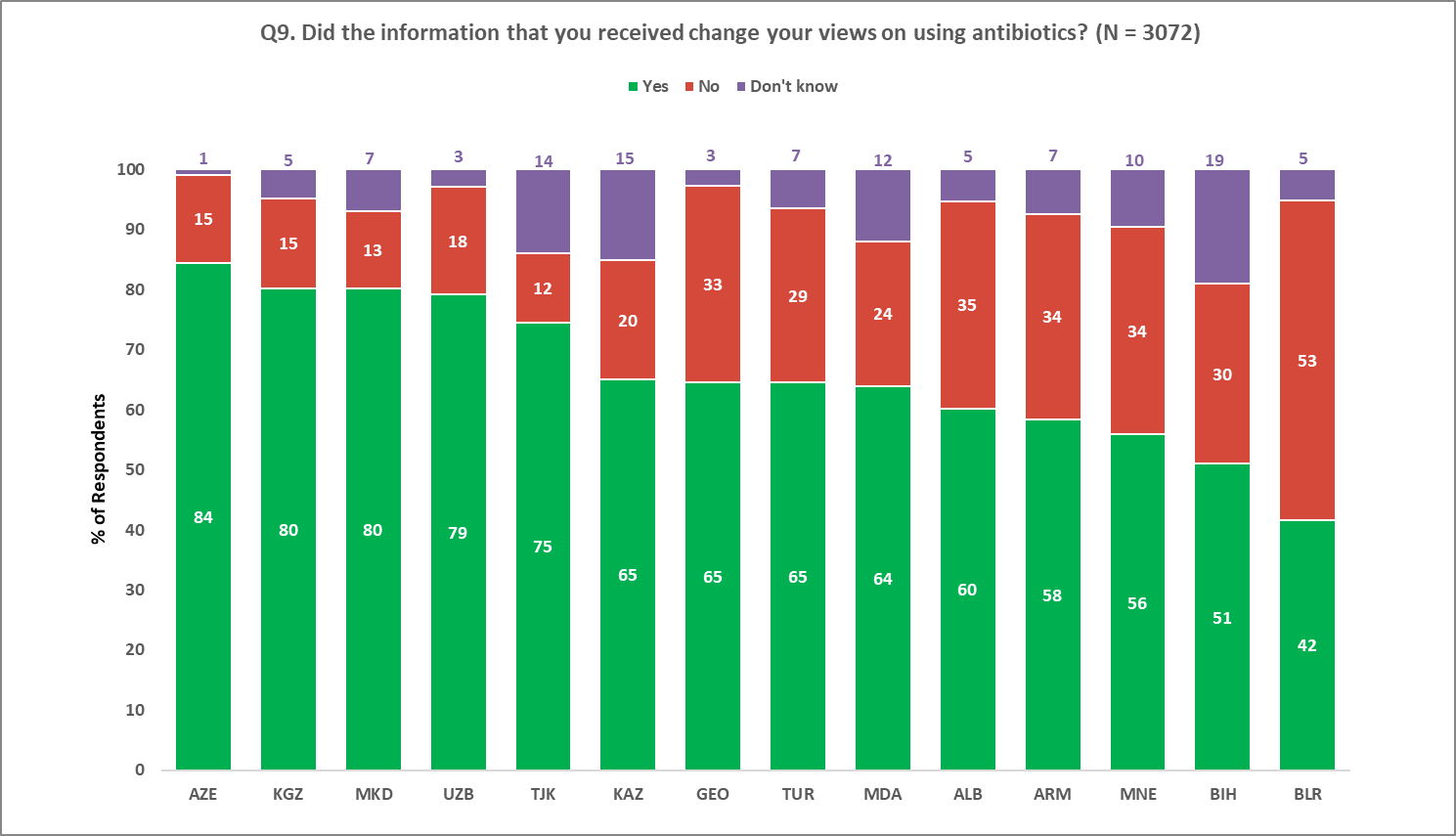


**Fig. 5b. Information leading to participants’ behaviour change concerning antibiotic use**

Note: Respondents were allowed to provide only one response for Q9.


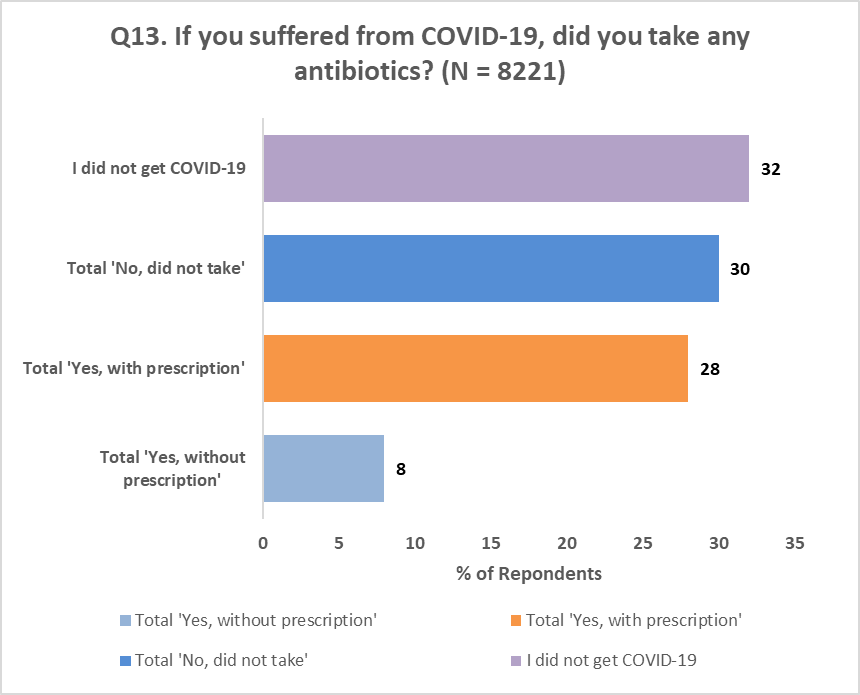


**Fig. 6. Use of antibiotics by the respondents for COVID-19**

Note: Respondents were allowed to provide multiple responses for Q13.


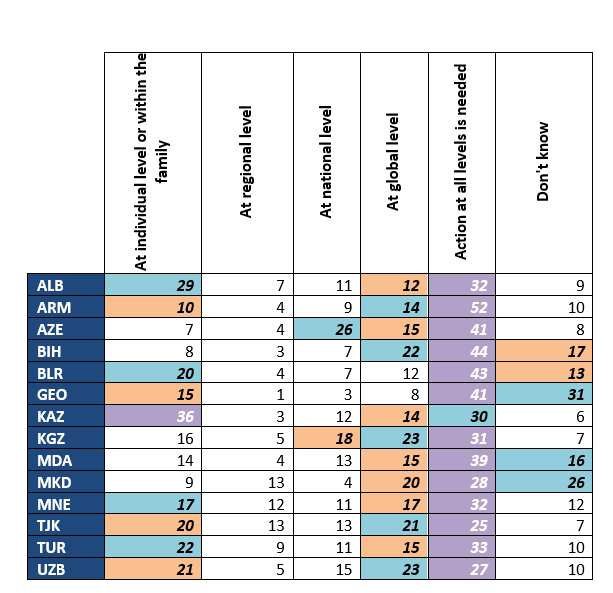


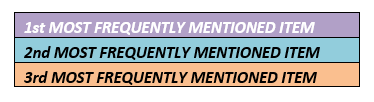


**Fig. 7. Respondents’ understanding of the correct level of policy intervention to tackle AMR**

Note: 1. All numbers are percentages of respondents.
2. Respondents were allowed to provide only one response for Q15.
